# Cys-loop receptors on cannabinoids: All high?

**DOI:** 10.3389/fphys.2022.1044575

**Published:** 2022-11-09

**Authors:** Philip Schmiedhofer, Florian Daniel Vogel, Filip Koniuszewski, Margot Ernst

**Affiliations:** ^1^ SBR Development Holding, Vienna, Austria; ^2^ Department of Pathobiology of the Nervous System, Center for Brain Research, Medical University Vienna, Vienna, Austria

**Keywords:** Cys-loop receptors, cannabinoid, epilepsy, chronic pain, cbd, thc, polypharmacy, polypharmacology

## Abstract

Endocannabinoids (eCBS) are endogenously derived lipid signaling molecules that serve as tissue hormones and interact with multiple targets, mostly within the endocannabinoid system (ECS). The ECS is a highly conserved regulatory system involved in homeostatic regulation, organ formation, and immunomodulation of chordates. The term “cannabinoid” evolved from the distinctive class of plant compounds found in *Cannabis sativa*, an ancient herb, due to their action on CB1 and CB2 receptors. CB1/2 receptors are the primary targets for eCBs, but their effects are not limited to the ECS. Due to the high interest and extensive research on the ECS, knowledge on its constituents and physiological role is substantial and still growing. Crosstalk and multiple targeting of molecules are common features of endogenous and plant compounds. Cannabimimetic molecules can be divided according to their origin, natural or synthetic, including phytocannabinoids (pCB’s) or synthetic cannabinoids (sCB’s). The endocannabinoid system (ECS) consists of receptors, transporters, enzymes, and signaling molecules. In this review, we focus on the effects of cannabinoids on Cys-loop receptors. Cys-loop receptors belong to the class of membrane-bound pentameric ligand gated ion channels, each family comprising multiple subunits. Mammalians possess GABA type A receptors (GABAAR), glycine receptors (GlyR), serotonin receptors type 3 (5-HT3R), and nicotinic acetylcholine receptors (nAChR). Several studies have shown different modulatory effects of CBs on multiple members of the Cys-loop receptor family. We highlight the existing knowledge, especially on subunits and protein domains with conserved binding sites for CBs and their possible pharmacological and physiological role in epilepsy and in chronic pain. We further discuss the potential for cannabinoids as first line treatments in epilepsy, chronic pain and other neuropsychiatric conditions, indicated by their polypharmacology and therapeutic profile.

## 1 Introduction

### 1.1 Cannabinoids

Cannabinoids are heavily studied polypharmacologically active molecules (Izzo et al., 2009), and can be classified as endocannabinoids (eCB’s), endocannabinoid-like mediators, and exogenous agents comprising phytocannabinoids (pCB’s) and synthetic cannabinoids (sCB’s) (Howlett et al., 2002; Di Marzo, 2020). Historically, they are called cannabinoids due to a class of phytochemicals, found in *Cannabis sativa L* (*Cannabacea*), that mimic some effects of the endogenous compounds (Martin et al., 1999; Gaoni and Mechoulam, 1964). The first identified and investigated endogenous cannabinoid, arachidonoyl ethanolamine or anandamide (AEA), derives from arachidonic acid (AA). AEA acts on the cannabinoid receptor 1 (CB1), the first identified classical cannabinoid receptor, inhibiting adenylate cyclase (Devane et al., 1992; Vogel et al., 1993). Various other endocannabinoids have been identified (Mechoulam et al., 1994; Howlett et al., 2002; Di Marzo et al., 2015). 2-AG has been first identified in 1995 and described as the major endocannabinoid (Mechoulam et al., 1995). 2-AG is a full agonist of CB1 and CB2 receptors, the latter having been described in 1993 (Munro et al., 1993). It occurs in higher concentration than AEA (Buczynski and Parsons, 2010). 2-AG is directly formed from diacylglycerol (DAG), a major second messenger in eukaryotic cells (Howlett, 2005; Di Marzo, 2008).

CB1 and CB2 receptors are both G-protein coupled receptors and expressed in various cell types and tissues throughout the body (Pertwee, 1997; Howlett, 2005; Joshi et al., 2020). Quantitatively, CB1 receptors are predominantly expressed in neurons, whereas CB2 receptors are primarily found on immune cells playing a substantial role in immunomodulation (Howlett, 2005). As newer findings indicate, both CB1 and CB2 receptors are expressed in neurons and both are involved in higher neuronal functions (Visvanathar et al., 2022). CB1 receptors are the most abundant GPR’s in the central nervous system (Baggelaar et al., 2018). Interestingly, they are found intracellularly on organelle membranes, such as mitochondria, presumably regulating cellular energy levels (Mendizabal-Zubiaga et al., 2016). Endocannabinoids can act in a retrograde fashion. This means that the endocannabinoids produced and released from the postsynaptic terminal into the synaptic cleft act on the presynaptic membrane. Presynaptic CB1 receptors regulate calcium release in the presynaptic terminal and thereby limit neurotransmitter release. This results in depolarization induced suppression of inhibition in case of GABA-ergic synapses (Alger 1999; Yoshida et al., 2002; Diana and Marty 2004).

The endocannabinoid system (ECS), consisting of receptors, enzymes and ligands, is involved in a variety of physiological processes, and critical to homeostasis (Behl et al., 2022). Endocannabinoid effects are not limited to targets associated with the endocannabinoid system (ECS). Crosstalk between various endogenous compounds and cannabinoid receptors have been described (Howlett et al., 2002; Pertwee et al., 2010). Endocannabinoids are important during neuronal development and pathogenesis of the nervous system (Harkany et al., 2008). Since research in the ECS is relatively young, their features and components are still heavily investigated. Researchers have thus postulated the term “endocannabinoidome” because orphan proteins and biomolecules, as well as existing components of other systems are being reclassified, expanding the ECS (Di Marzo et al., 2015; Di Marzo, 2020).

**FIGURE 1:**
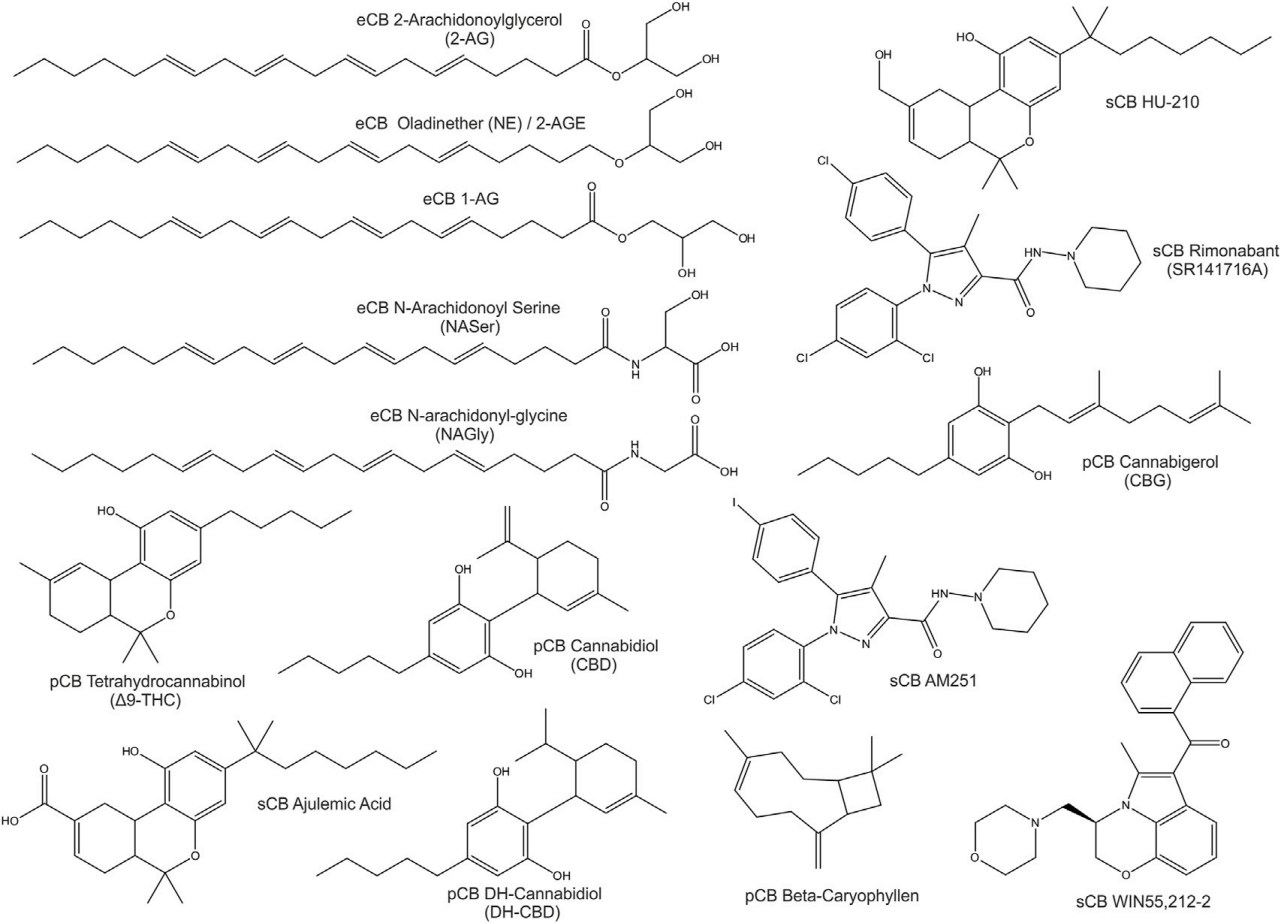
2D-Structures of selected cannabinoids: endogenous cannabinoids (eCB), phytocannabinoids (pCB) and synthetic cannabinoids (sCB).

Compounds of natural origin acting on the ECS (further referred as phytocannabinoids, pCB’s) are found in different plant and fungi species such as *Cannabis Sativa, Rhododendron, Echinacea angustifolia, Radula, Helichrysum umbraculigerum, Amorpha fruticosa, Glycyrrhiza foetida, Albatrellus, Cylindrocarpon olidum* (Raduner et al., 2006; Arif et al., 2021). Still, the term “cannabinoid” persists in use. “Cannabinoid” thus refers to compounds that act on an expanding system - the “endocannabinoidome”. Besides specific cannabinoid effects, the endocannabinoid system is externally influenced by epigenetics, feeding, lifestyle and clinical interventions (McPartland et al., 2014; Meccariello et al., 2020; Cavalli and Dutra, 2021). Representative eCBs, pCBs and sCBs are displayed in Figure 1.

Endocannabinoids derive from fatty acids. The major endocannabinoids derive from omega-6 unsaturated fatty acids such as arachidonic acid. Furthermore, some endocannabinoids derive from omega-3 fatty acids like EPA (eicosapentaenoic acid) and DHA (docosahexaenoic acid). However, both are constituents of phospholipid membranes and act as precursors for signaling molecules. Some are referred to as eicosanoids, like prostaglandins and leukotrienes (Howlett et al., 2002; Komarnytsky et al., 2021).

In contrast, classical phytocannabinoids such as tetrahydrocannabinol (Δ9-THC) and cannabidiol (CBD) derive from monoterpenoid compounds termed aminoalkylindoles, with poorly understood function within plants (Howlett et al., 2002).

Phytocannabinoids and endocannabinoids are lipophilic compounds, which is important to signaling functionality. Most cannabinoids and also the most abundant pCB’s such as CBD and Δ9-THC exert their effects *via* multiple molecular targets. The sCBs can be based on eCB or pCB structure.

#### 1.1.1 Target space of cannabinoids

Both endocannabinoids and phytocannabinoids were shown to interact with multiple distinct molecular targets. Very much like other plant compounds, phytocannabinoids influence many targets unrelated to CB1/2, and thus are perceived as “dirty drugs”. The first identified and most studied phytocannabinoid is Δ9-THC. Δ9-THC is mainly considered a potent partial agonist of CB1 and CB2 receptors. In addition, Δ9-THC is an agonist of the GPR18 receptor (McHugh et al., 2012, 2014). Δ9-THC has positive allosteric modulatory (PAM) effects on GlyRs (Hejazi et al., 2006; Xiong et al., 2012), and both positive and negative (PAM and NAM) effects on distinct GABAA receptors (Sigel et al., 2011; Schmiedhofer, 2017). Δ9-THC shows agonistic properties on TRPV2, TRPV3 and TRPV4 receptors (Qin et al., 2008; De Petrocellis et al., 2011, 2012), agonistic effects on PPARγ (O’Sullivan et al., 2006; Vara et al., 2013) and modulatory effects in opioid receptors (Kathmann et al., 2006). Furthermore, antagonistic properties were described (Barann et al., 2002; Shi et al., 2012; Morales et al., 2017) on 5-HT3A receptors and TRPM8 receptors (De Petrocellis et al., 2008).

Over the last decade, the most abundant phytocannabinoid in cannabis sativa, CBD, has been heavily studied. Interestingly, CBD is acting on more than 80 targets across various groups of receptors, transporters and enzymes. In contrast to Δ9-THC, it is devoid of psychotropic effects. This molecule’s target space has been studied and reviewed extensively (Izzo et al., 2009; Pertwee et al., 2010; Ibeas Bih et al., 2015) and is still growing. Among many other targets, CBD has been identified as an antagonist of the GPR55 receptor, now also known as CB3 receptor, which is so far unique for phytocannabinoids. CBD is devoid of effects on CB1/2 receptors, but antagonizes their agonists. This led to the hypothesis that it might be either a weak partial agonist, or a modulator of CB1/2 receptors (Pertwee, 2008). CBD has been suggested to exert anxiolytic and antiepileptic effects *via* 5-HT1A receptors, GlyRs and GABAARs, and some analgesic effects *via* TRP channels, but as will be discussed here, each endpoint likely involves multiple targets.

In summary, targets for cannabinoids comprise receptors, transporters and enzymes. Major targets are GPR’s such as CB receptors (CB1, CB2 and CB3), 5-HT1A receptors, adenosine receptors A1/A2 and opioid receptors. Furthermore, ion channels from multiple families, including the Cys-loop receptors and members of the TRP ion channels, are targets of pharmacological relevance. In addition, PPARγ, various enzymes involved in the synthesis and in the degradation of cannabinoids such as FAAH or COX-2, and CYP450 liver enzymes involved in the metabolism of various drugs are affected by cannabinoids. Furthermore, cannabinoids act on transporters and are involved in the uptake of dopamine, adenosine, glutamate and other signaling molecules (Izzo et al., 2009; Pertwee et al., 2010; Ibeas Bih et al., 2015; Cifelli et al., 2020).

## 2 Cys-loop receptors have cannabinoid interaction sites

This review provides an overview and a gap analysis on interactions of cannabinoids with Cys-loop receptors. Pharmacological and structural findings are summarized and framed by known and putative links between these targets and clinical indications for cannabinoid-based therapeutics. Furthermore, it should pose open questions, necessary to fill with future findings. Further experimental research clarifying molecular target identity and action, drug formulation, clinical investigation, and clinical intervention is anticipated.

In humans the term “Cys-loop receptors” refers to all pentameric ligand gated ion channels that are formed by subunits encoded by GABR, GLYR, CHRN, 5HT3 and ZAC genes (Koniuszewski et al., 2022). They form pentameric ligand gated ion channels known as cation selective nicotinic ACh receptors (nAChRs) and 5HT type 3 receptors (5-HT3Rs), the zinc activated cation channel (ZAC), and the anion selective GABA type A (GABAARs) and glycine receptors (GlyRs). Due to the highly conserved sequence and structure properties in this family, they share not only structural features but also ligands (Koniuszewski et al., 2022). While the agonist sites in the extracellular domain are less promiscuous, certain allosteric binding sites and specifically those in the transmembrane domain are quite similar across most Cys-loop receptors.

Some allosteric sites can confer agonist-like effects, but most elicit modulatory effects. Modulation refers to allosteric influences on the effects of the agonist, such as the widely known enhancement of GABA currents and increase in apparent GABA affinity induced by benzodiazepines for the case of GABAARs which is termed positive allosteric modulation (PAM). Silent and negative allosteric modulation can occur as well (SAM, NAM). We have recently analyzed the structural evidence for binding sites in human Cys-loop receptors (Koniuszewski et al., 2022 Figure 2) and provide here an update, with special focus on the known and candidate sites for cannabinoid interactions.

**FIGURE 2:**
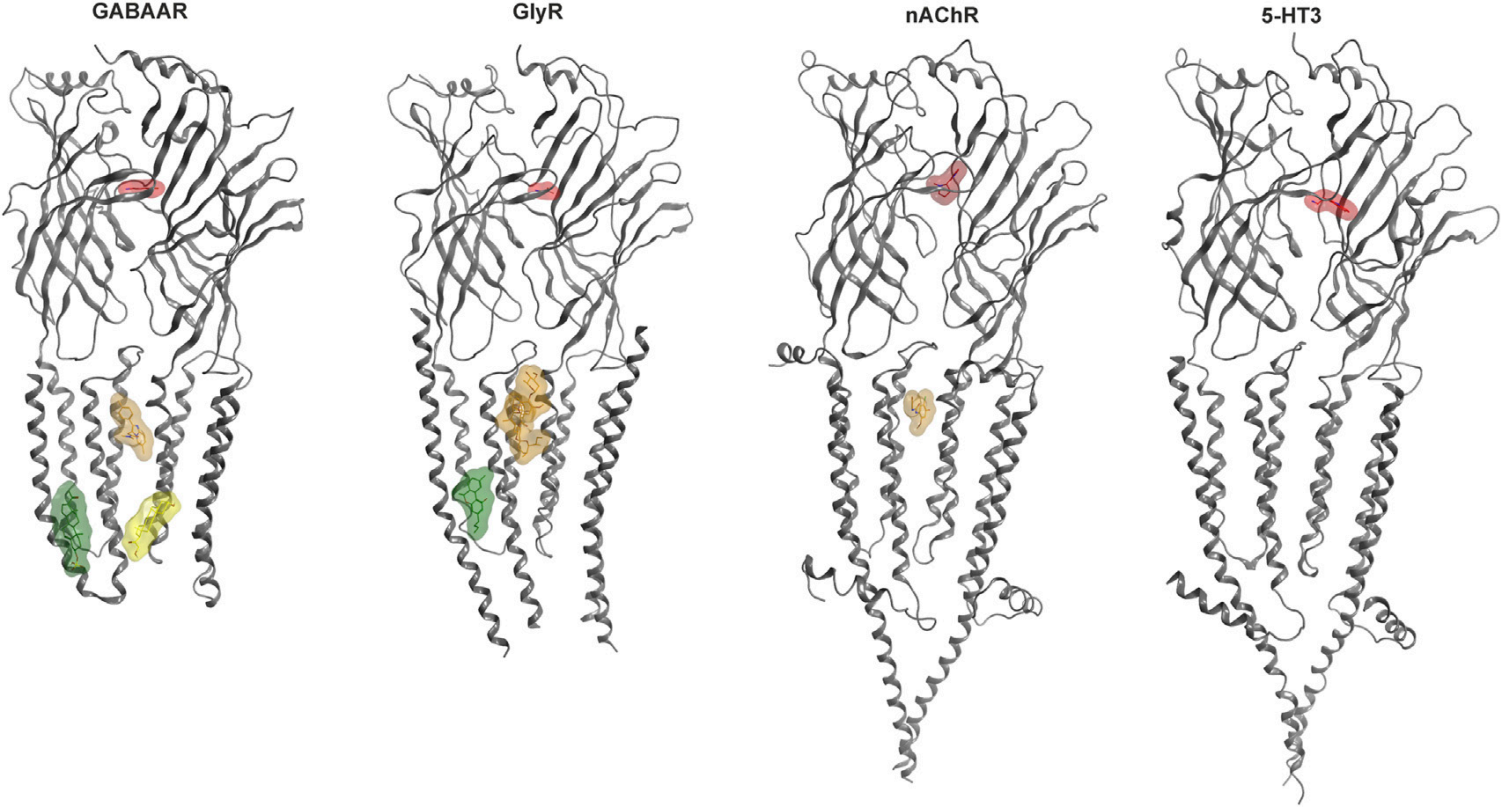
Cys-loop receptor structure and selected binding sites. For each family, a representative dimer is depicted in grey ribbon: GABAAR (8DD2—Zhu et al., 2022), GlyR (7M6O—Kumar et al., 2022), nAChR (7EKT—Zhao et al., 2021), 5-HT3 (6HIO—Polovinkin et al., 2018). Ligand bound structures have been superposed to render multiple sites. Agonist molecules at the ECD interfaces are rendered in red: GABA (PDB ID 6HUP—Masiulis et al., 2019), glycine (PDB ID 5VDH—Huang et al., 2017), nicotine (PDB ID 6PV7—Gharpure et al., 2019), 5-HT (PDB ID 6HIO). Binding sites in the TMD are displayed with representative ligands using colors for distinct sites: The upper TMD interface site in brown: zolpidem bound GABAAR (8DD2), ivermectin bound GlyR (5VDI - Huang et al., 2017) and PNU-120596 bound nAChR (7EKT). Lower TMD interface site in yellow: THDOC bound GABAAR (5OSB—Laverty et al., 2017). Lipid associated lower TMD site in green: pregnenolone sulfate bound GABAAR (5OSC—Laverty et al., 2017) and Δ9-THC bound GlyR (7M6O). The green ligand in the GlyR structure represents the first Δ9-THC bound Cys-loop receptors for which an atom level structure is available.

The GABAA receptor (GABAAR) assemblies are drawn from a panel of 19 mammalian subunits (six α, three β, three γ, one δ, three ρ, one ε, one π and one θ) and their respective variants (splice isoforms, RNA-editing variants). The channel formed by five subunits is anion selective. In neurons, they are ubiquitously present in synaptic and nonsynaptic (peri- and extra-synaptic) compartments (Olsen and Sieghart, 2008; Brickley and Mody, 2012), but also glial cell types and a wide range of non-neuronal cells express GABAARs. Many subunit assemblies are thought to consist of two α, two β and one γ subunits (Olsen and Sieghart, 2008), with recent evidence pointing to a wide diversity of alternative combinations (Sente et al., 2022). GABAAR targeting compounds interact either with orthosteric (GABA) sites or with allosteric sites, which are present in the extracellular and membrane spanning domains (Koniuszewski et al., 2022).

Early research focused on synaptic GABAA receptors in the CNS, as well as those in non-neuronal tissues. Neuronal extrasynaptic receptors, described more recently, are highly sensitive to ambient GABA concentrations and mediate chiefly tonic inhibition. Those receptors are composed mainly of α1, α4, α5, α6 and δ subunits along with β subunits, and display distinct pharmacology. They feature less or no desensitization, higher agonist affinity and lower agonist efficacy, and lack the canonical high affinity binding sites for benzodiazepines (Mody and Pearce, 2004; Farrant and Nusser, 2005; Mortensen and Smart, 2006; Zheleznova et al., 2009).

**FIGURE 3:**
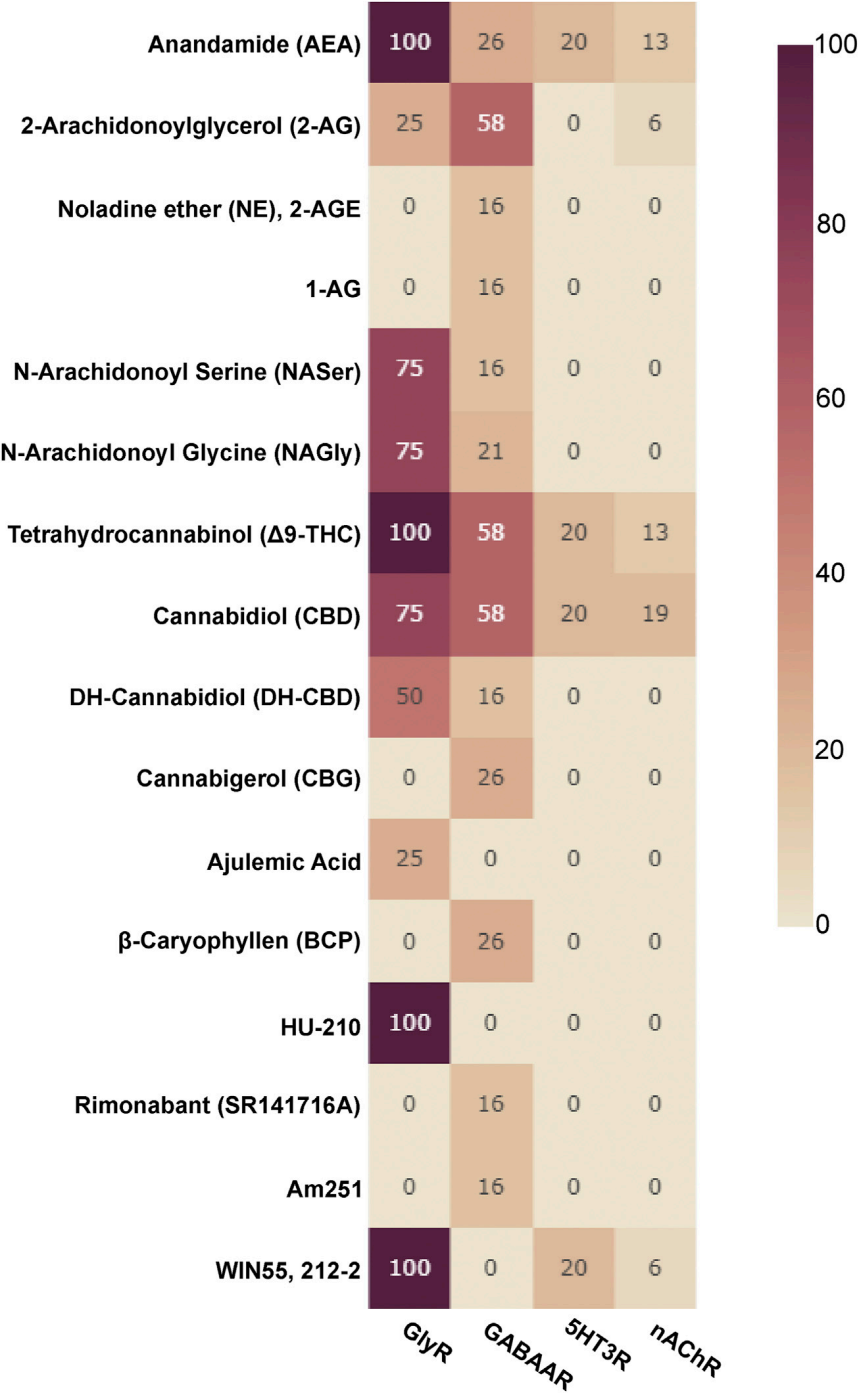
Cannabinoids tested on Cys-loop receptors: The fraction of subunits per Cys-loop family which have been tested with the listed cannabinoids is displayed as a heatmap (X of 19 for GABAARs, X of 16 for nAChRs, X of 4 for GlyRs and X of 5 for 5HT3Rs, no data is available for ZAC).

Allosteric compounds that enhance GABA effects exert chiefly CNS depressant actions such as sedation, and induce an increase of seizure threshold in anti-epileptic treatment. In contrast, reduction of GABA effects leads to CNS stimulatory effects such as arousal, anxiogenesis, and seizures. The extrasynaptic pool of GABAARs has raised attention as putative group of targets for a wide diversity of neuropsychiatric conditions in addition to the established indications of GABAAR targeting drugs as hypnotic-sedatives, anxiolytics and antiepileptics (Brickley and Mody, 2012). Cannabinoid effects have been observed for different GABAAR subtypes, see Figure 3 for an overview and Supplementary Table S1 for details. Interestingly, phytocannabinoids (Δ9-THC) can elicit effects consistent with either positive or negative modulation, depending on the β subunit incorporated (Schmiedhofer, 2017). Endocannabinoid modulation was demonstrated for β2-subunit containing GABAARs (Baur et al., 2013a; Baur et al., 2013b).

CBD has been shown to elicit moderate GABA enhancing (PAM) effects on α1β1, α2β1, α1β2, α2β2, α1β1γ2, α2β1γ2, α1β2γ2 and α2β2γ2 with higher effects on α2 containing receptors in injected *Xenopus laevis* oocytes, and also enhanced GABA currents in micro transplanted membranes of Dravet syndrome (DS) patients. These findings indicate a binding site, not affected by the γ subunit (in contrast to benzodiazepines), and an α selectivity in favor of α2, compared to the α 1, in α + β1/2 + γ2 containing receptor populations (Bakas et al., 2017; Ruffolo et al., 2018).

Furthermore, CBD shows strong positive modulatory effects on α4β2δ receptors (Bakas et al., 2017) and α4β3δ receptors (Schmiedhofer, 2017). Among the β isoforms, functional preference for β2 and β3 over β1 for CBD (Bakas et al., 2017) and similarly for Δ9-THC (Schmiedhofer, 2017) has been observed. Interestingly an incorporation of β1 subunits in δ containing subunit combinations results in negative allosteric modulation by CBD and Δ9-THC, as observed in a pilot study (Schmiedhofer, 2017). However, CBD discriminates against Δ9-THC in regard to the PAM effects on α4β3δ receptors but not in α6β3δ adding further specific properties to CBD compared to Δ9-THC (Schmiedhofer, 2017).

Glycine receptors (GlyRs) are a smaller family of pLG anion channels, featuring five human GlyR subunits (α1-4, β) (Lynch, 2004; Lynch et al., 2017). Similar to GABAA receptors, they chiefly mediate inhibitory effects in the CNS. In the spinal cord and brain stem they are mainly located at the post-synapse, whereas in the brain presynaptic and extrasynaptic GlyRs are more abundant (Lynch, 2004). GlyRs are discussed as potential targets for the treatment of pain (Xiong et al., 2012; Lu et al., 2018; Gradwell et al., 2020; Zeilhofer et al., 2021). CBD and Δ9-THC are potentiating glycine effects in native spinal neurons, as well as in recombinant α1 and α1β1 GlyRs (Xiong et al., 2011). Recently, a Δ9-THC- bound GlyR structure has been released (Kumar et al., 2022) which provides the first atom resolution insight into cannabinoid interaction sites at Cys-loop receptors. These findings complement and frame results from previous mutational studies. The cannabinoids AEA, 2-AG, Δ9-THC, CBD, NA-Gly, NA-Ser and HU-210 have been found to modulate GlyR differently, depending on the subunits incorporated. AEA, 2-AG, Δ9-THC and CBD show PAM effects on α1/α2/α3-GlyR, NA-Gly, NA-Ser and HU-210 show NAM effects in α2/α3-GlyR and PAM effects in α1-GlyR (Yang et al., 2008; Yévenes and Zeilhofer, 2011;).

Due to the high homology between GABAA- and Gly-receptors, the allosteric binding sites appear to be largely overlapping (Koniuszewski et al., 2022, and Figure 2). Prior to the recent structural findings, mutational studies have examined possible localizations of cannabinoid sites in Cys-loop receptors, see Table 1.

**TABLE 1:** Mutational analysis of candidate binding sites.

Amino acid numbering in Figure 2	Glycine receptors	
1	GlyR α1/3 subunits S296A—decreased potentiation for AEA	Xiong et al. (2012)
	A303S of the α2 subunit significantly increased the magnitude of AEA-induced potentiation	
1	Direct interaction between CBD and S296 in the TMD3 of purified GlyR-α3	Xiong et al. (2012)
1	S296 is a critical site for DH-CBD potentiation of α1 GlyRs	Lu et al. (2018)
1	S296 in hGlyR-α1 is dependent for Δ9-THC effects	Wells et al. (2015)
2	GlyR α1 S267I in TM2 - diminishes effects of ajulemic acid, cannabidiol and HU210	Foadi et al. (2010)
2	GlyR α subunits S267Q - no binding site for AEA or Δ9-THC	Hejazi et al. (2006)
	the S267Q substitution did not change the current enhancement induced by Δ9-THC or AEA	
3	DH-CBD at 10 μM significantly reduced the glycine EC50 value and increased the maximal IGly value for the α1 R271Q GlyRs	Xiong et al. (2014)
1	S296A in GlyR-α1 significantly reduced the GlyR current potentiation by NA-Gly	Yévenes and Zeilhofer (2011)
4	GlyR-α1/α2/α3 K385A were significantly less potentiated by AEA	
	K385 residue is critical for the PAM of all the GlyR isoforms by both acidic and neutral eCB derivatives, but appears to be dispensable for the inhibitory actions of acidic eCB on α2 and α3 GlyRs	
5	I240V in GlyR-α1 significantly reduced the GlyR current potentiation by NA-Gly	
6	G254A in GlyR-α1 significantly reduced the GlyR current potentiation by NA-Gly	
	**GABAA receptors**	
-	α1β2N265Sγ2 effects of AM251 were decreased to ∼50%	Baur et al. (2012)
	α1β2N265Sγ2 (loreclezol binding site) effects of AM251 were still increased by 50%	
-	α1β2V436Tγ2 effects of 2-AG were decreased to 4% ± 16	Baur et al. (2013b)
	α1β2F439Lγ2 effects of 2-AG were decreased to 31% ± 10	
	α1β2M294Lγ2 effects of 2-AG were decreased to 36% ± 15	
	α1β2V302Cγ2 effects of 2-AG were decreased to 43% ± 18	
	α1β2W428Cγ2 effects of 2-AG were decreased to 44% ± 15	
	α1β2L301Fγ2 effects of 2-AG were decreased to 45% ± 18	
	α1β2V443Cγ2 effects of 2-AG were decreased to 49% ± 13	
	α1β2S429Cγ2 effects of 2-AG were decreased t 49% ± 13	
	α1β2F432Cγ2 effects of 2-AG were decreased to 63% ± 18	
	α1β2I305Cγ2 effects of 2-AG were decreased to 77% ± 8	
	α1β2R427Cγ2 effects of 2-AG were decreased to 78% ± 10	
	α1β2I305Cγ2 effects of 2-AG were decreased to 84% ± 9	
	α1β2F440Cγ2 effects of 2-AG were decreased to 98% ± 19	
	α1β2V436Cγ2 effects of 2-AG were decreased to 99% ± 27	
	α1β2I442Cγ2 effects of 2-AG were increased to 101% ± 28	
	α1β2L301Cγ2 effects of 2-AG were increased to 101% ± 44	
	α1β2V435Cγ2 effects of 2-AG were increased to 101% ± 17	
	α1β2L446Cγ2 effects of 2-AG were increased to 102% ± 20	
	α1β2R430Cγ2 effects of 2-AG were increased to 102% ± 43	
	α1β2N441Cγ2 effects of 2-AG were increased to 103% ± 26	
	α1β2Y447Cγ2 effects of 2-AG were increased to 104% ± 19	
	α1β2I425Cγ2 effects of 2-AG were increased to 105% ± 35	
	α1β2S438Cγ2 effects of 2-AG were increased to 106% ± 34	
	α1β2F439Cγ2 effects of 2-AG were increased to 107% ± 32	
	α1β2N423Cγ2 effects of 2-AG were increased to 112% ± 38	
	α1β2I431Cγ2 effects of 2-AG were increased to 113% ± 32	
	α1β2A424Cγ2 effects of 2-AG were increased to 116% ± 27	
	α1β2Y444Cγ2 effects of 2-AG were increased to 120% ± 27	
	α1β2W445Cγ2 effects of 2-AG were increased to 134% ± 46	
-	effects of CBD and 2-AG at α2β2V436Tγ2L receptor subtypes were reduced to levels similar to that seen at α2β1γ2L receptors	Bakas et al. (2017)
-	α1β2W428Cγ2 largely abrogates modulation by NA-glycine after 1 min of combined application of GABA with NA-glycine	Baur et al. (2013a)
	α1β2S429Cγ2 largely abrogates modulation by NA-glycine after 1 min of combined application of GABA with NA-glycine	
	α1β2F439Lγ2 largely abrogates modulation by NA-glycine after 1 min of combined application of GABA with NA-glycine	
	α1β2V443Cγ2 largely abrogates modulation by NA-glycine after 1 min of combined application of GABA with NA-glycine	

nAChRs, 5-HT3Rs and ZAC, are pLG cation channels which share high homology with each other, and lower homology with the anion channels (Koniuszewski et al., 2022).

16 nAChR subunits (α1-7, α9,10, β1-4, γ, δ and ε) are available to form pentameric assemblies around a central sodium- and calcium-selective pore. Muscle specific and neuron specific heteromeric and homopentameric nAChRs have been described (Gotti et al., 2006; Gotti et al., 2007; Zoli et al., 2018). Neuronal nAChRs occur in the central and the peripheral nervous system, and at the neuromuscular junction, the muscle-type is found. The peripheral subtypes (mainly found in the autonomic nervous system) as well as the muscle type are targets of a wide range of medications. Here we are mostly interested in the “brain-type” nAChRs. Brain nAChRs are implicated in various diseases and disorders including nicotine addiction, epilepsy, Parkinson’s and Alzheimer’s diseases (Corradi and Bouzat, 2016; Scholze and Huck, 2020; Wu et al., 2021). Smoking cessation aids target the brain type receptors. In the case of varenicline, agonism and partial agonism are known at α7-homomers and at α4β2 heteropentamers, respectively (Mihalak et al., 2006). 2-AG, AEA and methanandamide (mAEA), a stable analog of AEA, had been identified as inhibitors of α7-AChR (Oz et al., 2003; Oz et al., 2004; Baranowska et al., 2008). CBD has been identified as NAM of α7-nAChRs at micromolar concentrations in whole-cell patch clamp recordings in rat hippocampal slices (Mahgoub et al., 2013), while Δ9-THC is relatively ineffective at inhibiting α7-nAChRs. α4β2-nAChR are inhibited by AEA (Spivak et al., 2007; Butt et al., 2008).

Five 5-HT3Rs subunits are known (namely A, B, C, D and E) (Tzvetkov et al., 2007; Walstab et al., 2010; Brüss et al., 2000) and a single human gene encodes the zinc-activated channel (ZAC) (Davies et al., 2003). 5-HT3ARs are the target of many antiemetics. Interestingly, a new line of research is investigating the usefulness of 5HT3R negative allosteric modulators in the cure of irritable bowel syndrome (Farthing, 1999). Δ9-THC inhibits 5-HT3Rs with similar potency to CB1 receptors (Barann et al., 2002; Oz et al., 2004). CBD has been identified as a negative allosteric modulator (NAM) in 5-HT3Rs receptors in *Xenopus laevis* oocytes and HEK cells respectively (Yang et al., 2010b; Xiong et al., 2011). The effects at ionotropic 5HTRs are thought to contribute to anti-emetic and anti-inflammatory action. Figure 3 summarize the body of literature in which the effects of cannabinoids on diverse Cys-loop receptors have been investigated.

**FIGURE 4:**
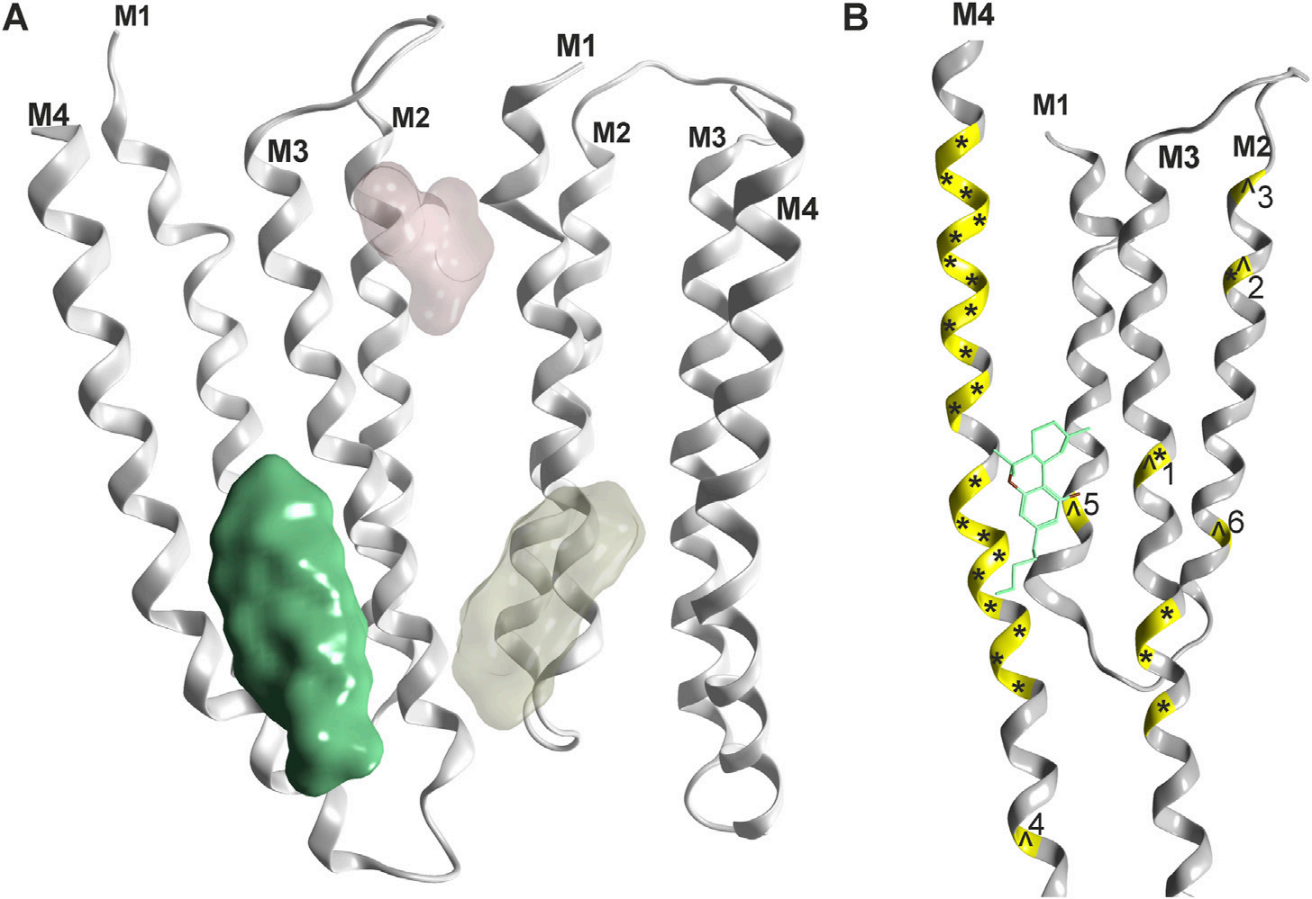
Phytocannabinoid sites on Cys-loop receptors: **(A)** Overview, one confirmed site (green, rendering of 7M6Q Kumar et al.) and two superposed candidate sites based on mutational evidence and computational docking (Koniuszewski et al., 2022, yellowish and light red). **(B)** Detailed rendering of 7M6Q with Δ9-THC bound. Mutational sites as shown in Table 1 are color coded on the ribbon in yellow. Mutations in the glycine receptor are marked with an ^ and mutations in the GABAA receptor are marked with an *. Numbers refer to Table 1 for individual mutated sites.

For all Cys-loop receptors that form heteropentameric assemblies, the pharmacological testing of compound effects poses specific challenges related to the ill defined pentamer composition, stoichiometry and arrangement that might occur in recombinant expression systems. An illustrative example is the recent observation of multiple distinctive pentamers that are formed upon co-expression of three GABAAR subunits in HEK cells (Sente et al., 2022). Despite this limitation, several studies utilizing wild type and mutated subunits of Cys-loop receptors investigated candidate binding sites for cannabinoid molecules, see Figure 4 and Table 1. For the case of α1-GlyRs, the site previously proposed (Xiong et al., 2012) has recently been confirmed with electron microscopy (Kumar et al., 2022).

### 2.1 Multiple allosteric sites: Complex allosteric interactions

It is very gratifying to note that the confirmed binding site for Δ9-THC (Kumar et al., 2022) indeed is localized in one of the proposed positions (Xiong et al., 2011). This site corresponds topologically with the candidate site for endocannabinoids in the GABAAR β2 subunit (Baur et al., 2013a). Mutational analysis suggests additional cannabinoid sites in α1-GlyRs at the upper TMD interface (Foadi et al., 2010 light red site in Figure 4), and the recent structural work reports unclear densities in this region as well (Kumar et al., 2022). Future studies will show which subunits of the entire Cys-loop receptor family share this specific site, by which molecules it is used, and if some of the other sites that have been proposed are also sites for cannabinoid molecules. So far, binding sites in the TMD utilized by lipophilic molecules largely overlap with sites, at which lipid constituent molecules such as cholesterol have been observed as well (see Supplementary Figure S1 and Supplementary Figure S2). This has precipitated the use of the term “lipid-associated binding sites” for interaction sites which are not protein pockets in the classical sense, but appear to be formed by “grooves” in the TMD together with the lipid collar.

Synergistic effects such as additive, supra-additive or infra-additive effects can occur when multiple compounds affect multiple binding sites (Hoyt et al., 2022). Nonlinear allosteric additivity can be observed in co-application experiments with multiple compounds, or in the presence of endogenous substances. This has been shown with the combination of the neurosteroid pregnenolone and the benzodiazepine triazolam (Fischer and Rowlett, 2011; Gunter et al., 2015), and for allopregnanolone and etomidate (Li et al., 2014). Furthermore (Sigel et al., 2011) showed a supra-additive effect between 2-AG and the neurosteroid THDOC (3α, 21-dihydroxy-5α pregnan-20-one), and 2-AG and diazepam respectively. Moreover, membrane cholesterol interacts with glycine receptors, and is required for Δ9-THC modulatory effects, but not in 5-HT3A receptors or GABAAR subtype α1β2γ2, as shown in HEK293 cells and cultured spinal neurons (Yao et al., 2020).

Pilot studies indicated that complex mixtures of more than one cannabinoid had greater effects than those individually applied. Moreover (weak) effects can be masked due to secondary plant compounds (probably terpenes and flavonoids), as shown in Supplementary Figure S3 (Schmiedhofer, 2017). Secondary plant compounds can have modulatory effects on Cys-loop receptors (Nilsson and Sterner, 2011; Kessler et al., 2014; Sucher and Carles, 2015; Milanos et al., 2017). Thus, to clarify the effects of plant extracts and their constituents individually, there is a need for more experimental research, adding compounds to endogenous substances such as steroids, or endocannabinoids, to explore and quantify additive or synergistic (supra- or infra-additive) effects.

Generally, each Cys-loop receptor pentamer contains multiple allosteric sites, which can be occupied simultaneously by identical or different molecules. Furthermore, conformational changes are induced by ligands and impact globally on the receptor properties and other binding sites (Koniuszewski et al., 2022). This leads to an enormous range of possible molecular synergistic effects and makes the *in vitro* effects of individual compounds alone questionable—a compound which enhances GABA elicited currents alone might lower the net response to GABA and eCBs, for example. Thus, the terms PAM and NAM may suggest more insight into a compound effect than the current state of knowledge can offer. A more structured experimental approach, especially in regard to subunit expression, dose, and co-application of compounds is needed.

## 3 Cannabinoids in recreational and clinical use

The spectrum of cannabinoids consumed by humans is mostly plant derived, chiefly from cannabis plants (flowers or leaves, often just called “cannabis”) but also from *Echinacea* and other plant sources. Moreover, synthetic compounds are available, often referred to as research chemicals, if not approved as API (active pharmaceutical ingredient). Most cannabinoid use, whether for recreational or medical purposes, occurs in non-clinical settings. Natural cannabis is considered the most commonly used drug in the world and has been used for centuries. In addition to the use of cannabis flowers and extracts, the use of tinctures, particularly those containing CBD which are often produced from industrial hemp, has been a widespread trend over the past decade. Anecdotal information from cannabis users guides clinical research and applications, and *vice versa*. Due to the stigma of cannabis and the implementation of strict legal concepts at the UN level in the 1970s, today’s regulatory developments have led to complex legal situations. In most countries, oral use of cannabis products is restricted even when smoking of these products is regulated, and in some places considered legal. Cannabis research had been suppressed for a long time, but the last decades revealed unprecedented data, and questions the strict scheduling of cannabis as a substance without medical use. Cannabis use is considered safe among other scheduled drugs. However, besides the recreational use of cannabinoids and despite complex legal situations that differ across countries and regulatory bodies, clinical use is on the rise.

### 3.1 Medicinal cannabinoid preparations

To illustrate the complexity of cannabinoid medicines, a short overview of cannabinoid varieties in medical use and in clinical research is provided in Table 2. We need to distinguish between single compound active pharmaceutical ingredient(s) (API) and plant extracts as API, used in both proprietary pharmaceuticals or magistral preparations. On top of that, medical cannabis has been defined and usually means flowers from cannabis plants. Different strains of cannabis plants exist, but plants are best described as defined chemovars with defined contents of phytocannabinoids and terpenes (Lewis et al., 2018).

**TABLE 2:** Cannabinoid preparations and their respective source materials, overview.

Single compounds	Plant extracts	Medical cannabis
CBD (API)	Δ9-THC rich extracts as API and/or CBD rich extracts as API	chemovars of cannabis flowers as API
• Epidiolex^®^	• Sativex^®^ (Nabiximols^®^)	
Δ9-THC (Dronabinol^®^) (API)		classified by
• Syndros^®^	other extracts	• certain cannabinoids
• Marinol^®^	• minor cannabinoid enriched extracts - e.g CBG extracts	• terpenes
Nabilon (API) (Cesamet^®^, Canemes^®^)		
Rimonabant^®^ (SR141716A) (API)		
synthetic cannabinoids (research chemicals)		

Magistral formulations are a common method to fill prescriptions. Doctors can prescribe substances in different application forms (tinctures, topicals, capsules, suppositories, whole plant parts — flowers, in case of cannabis) and pharmacies prepare them individually for patients, using raw plant materials, plant extracts or isolated cannabinoids in the respective vehicles. In the case of single compounds, in their natural or synthetic form, simple regulations apply. This makes them easier to compare in clinical applications, due to their consistency, which is not the case for whole plant preparations. There are plenty of new chemovars of cannabis available as API, which show distinct active ingredients. This could be of advantage to patients, because individuals can finetune effects. On the downside, the diversity and variability of natural products complicates the collection and interpretation of clinical observations.

There are differences between approved pharmaceutical products and natural plant materials, even if their main active ingredients are the same. Because some methods (extraction, purification, improving shelf life, reduction of impurities and radiation) alter the natural composition of plants, the direct comparison between medical and raw cannabis is not scientifically valid.

At this time though, most *in vitro* studies, preclinical, *in vivo*, and clinical evidence has been accumulated for the use of single compounds, either CBD or Δ9-THC, or synthetic cannabinoids, or combinations of a few phytocannabinoids. As doctors prescribe more cannabis flowers, more clinical data on whole plant products will be available in the near future.

### 3.2 Approved medicinal products

There are several cannabinoid based pharmaceutical prescription drugs, approved by FDA, MRHA or EMA, with both natural or synthetic origin, listed in historical order in Table 3. In addition, a variety of magistral preparations are available. Nabilone (Cesamet^®^, Canemes^®^) was patented in 1975 (Lilly) and was the first synthetic derivative of Δ9-THC. The natural compound Δ9-THC (Dronabinol^®^) is used in magistral preparations and in capsules (Marinol^®^) or as a solution (Syndros^®^). Δ9-THC rich extracts (including minor cannabinoids and other plant compounds) are used in combination with a CBD rich second extract in a proprietary pharmaceutical product called Sativex^®^ (Nabiximols^®^). This is a milestone and shows the possibility of using whole plant extracts in proprietary pharmaceutical products. However, the production of whole plant extracts according to pharmaceutical guidelines is difficult compared to the use of purified compounds. Natural CBD, in its purified form, is used in Epidiolex^®^ (Epidyolex^®^) and magistral preparations (various applications such as tinctures, topicals, suppositories and capsules), and available as OTC products (CANNEFF^®^ SUP suppositories, EU-registered medical device). Furthermore, various OTC products and further proprietary pharmaceutical products are currently under development, lining up for market approval. As mentioned above, cannabis flowers are active pharmaceutical ingredients (APIs). Therefore, extracts prepared from these whole plant parts are also considered APIs and can be used in magistral preparations. The indications of those approved pharmaceutical products and medical devices are listed in Table 3.

**TABLE 3:** Approved cannabinoid medications and their active ingredients, indications, and product types. Meta analysis findings from (Bilbao and Spanagel, 2022) are included for “high” and “moderate” evidence indications.

API	Drug	Indications	Product type
C24H36O3, Nabilone is a racemic mixture consisting of (S,S)-(+)- and (R,R)-(−)-isomers of a synthetic derivative of Δ9-THC	Nabilone	Nausea and vomiting associated with cancer chemotherapy in patients who have failed to respond adequately to conventional antiemetic treatments	Pharmaceutical (FDA, EMA, MRHA)
	Cesamet^®^		
	Canemes^®^		
Δ9-THC	Dronabinol^®^	Anorexia associated with weight loss in patients with AIDS. Nausea and vomiting associated with cancer chemotherapy in patients who have failed to respond adequately to conventional antiemetic treatments	Active Pharmaceutical Ingredient for magistral preparations (Dronabinol^®^)
	Marinol^®^(Δ^9^-trans-tetrahydrocannabinol)	Evidence from meta-analysis: Moderate evidence: Chronic pain, appetite, Tourette Syndrome (Bilbao and Spanagel 2022)	Capsules (Marinol^®^ - FDA)
	Syndros^®^		Δ9-THC Solution (Syndros^®^ - FDA)
extract containing Δ9-THC and extract containing CBD	Sativex^®^	Severe spasticity due to multiple sclerosis (MS)	Pharmaceutical: (FDA, EMA, MRHA)
	Nabiximols^®^	Evidence from meta-analysis: Moderate evidence: Chronic pain, spasticity, sleep, substance use disorders (SUDs) (Bilbao and Spanagel 2022)	
CBD	Epidiolex^®^	Seizures Associated with Lennox-Gastaut Syndrome or Dravet Syndrome	Pharmaceutical
	Epidyolex^®^	Seizures Associated with Tuberous Sclerosis Complex	Epidiolex^®^ (FDA) Epidyolex^®^ (EMA, MRHA)
	CBD API	Evidence from meta-analysis: High evidence: epilepsy, Moderate evidence: parkinsonism (Bilbao and Spanagel 2022)	CBD API magistral preparations
CBD + Hyaluronic acid	CANNEFF^®^ SUP/VAG SUP	vaginal dryness, vaginal discomfort, painful intercourse, vaginal dystrophy, nonspecific inflammation of the intestines, fissures and lesions, hemorrhoids, proctitis	Suppositories, medical device

There are various indications under clinical investigation, and some are already in off-label use. In total, there is already plenty of evidence of clinical effectiveness of cannabinoids, as well as for the safe use of phytocannabinoids in humans (Zeraatkar et al., 2022). According to the latest systematic reviews and meta-analysis for all relevant indications, the drugs Dronabinol^®^, nabilone, cannabidiol and Sativex^®^, have the following relevance as therapeutic tools in medicine (Table 3): Dronabinol^®^ was moderately effective in chronic pain, for inducing appetite, and in alleviating Tourette symptoms. CBD was found most effective in seizures and moderately effective in parkinsonism. Sativex^®^ was found moderately effective in chronic pain and spasticity, followed by improving sleep and SUD’s (substance abuse disorders) (Bahji et al., 2022; Bilbao and Spanagel, 2022). For all other indications, this meta-analysis (Bilbao and Spanagel, 2022) reports low or very low evidence, but not all trials could be included due to methodological differences.

Ongoing clinical trials were identified by a database search (active, not recruiting – clinical trials.gov) and are listed in Table 4.

**TABLE 4:** Ongoing clinical trials using CBD or Δ9-THC, according to a database search (active, not recruiting) at clinical trials.gov.

Search	Trials (grouped by indication)
CBD	Epilepsies: Cannabidiol in Children with Refractory Epileptic Encephalopathy (CARE-E) (NCT03024827)
	A Double-Blind Trial to Evaluate Efficacy and Safety of Cannabidiol as an add-on Therapy for Treatment in Refractory Epilepsy (NCT02783092)
	Psychotic disorders: Enhancing Recovery in Early Schizophrenia (NCT02926859)
	A Four-week Clinical Trial Investigating Efficacy and Safety of Cannabidiol as a Treatment for Acutely Ill Schizophrenic Patients (NCT02088060)
	CBD Cigarettes Instead of Normal Cigarettes as Innovative Treatment for Schizophrenia (NCT04700930)
	Cannabidiol Treatment in Patients with Early Psychosis (CBD) (NCT02504151)
Δ9-THC	Chronic Pain: Canadian Registry for the Use of Spectrum Therapeutics Cannabis Products in Subjects with Chronic Pain (NCT04763252)
	MEMO-Medical Marijuana and Opioids Study (NCT03268551)
	Anxiety/Depression/Insomnia/Pain: Effect of Medical Marijuana on Neurocognition and Escalation of Use (MMNE) (NCT03224468)
	Schizophrenia: Probing the Cannabinoid System in Individuals with a Family History of Psychosis (NCT02102113)
	Psychotic Disorders: Cannabinoids, Learning, and Memory (Δ9-THC-Memory) (NCT02407808)

As can be gleaned from the large number of completed trials as reviewed recently (Bilbao and Spanagel, 2022) and the growing body of literature, pCBs and selected sCBs have established their place in medical applications and can be expected to be used in many new indications in the near future. Presumably the effectiveness of various whole plant preparations will be confirmed in trials in the near future, as there is accumulating anecdotal evidence as well as from the literature for many indications.

### 3.3 Recent advances in dosing and application

Dosing of cannabinoids varies tremendously between preparations. Nowadays, daily dosages of 350–1750 mg pure CBD are common in epileptic patients, limiting the use for some patient groups with hepatic dysfunctions (Devinsky et al., 2021). Especially in epilepsies, where CBD is used as a registered drug (Epidiolex^®^) or in magistral preparations (Devinsky et al., 2016; Thiele et al., 2018), new data suggest possible superior effects of whole plant preparations (Goldstein, 2015; Pamplona et al., 2018). The prevention of convulsive status epilepticus shows direct influence on the improvement of seizure prognosis (Akiyama et al., 2010).

Furthermore, a recently published German survey conducted by Bfarm shows that the median daily intake of Δ9-THC varies enormously between Dronabinol^®^ (25 mg) and Flowers (249 mg). Flowers were most commonly used against pain (Bundesinstitut für Arzneimittel und Medizinprodukte, 2022).

Among all cannabinoid medicines on the market, there are differences in bioavailability between application forms, and preparations, and different dosing regimens. Dosing and application of cannabinoid preparations present issues in practical use (Notcutt et al., 2004) remaining unsolved yet. Several new application techniques raise hope, addressing dosing issues due to more efficient different application methods in the future. Particularly with new forms of administration with rapid onset of action and higher bioavailability, such as encapsulated and breathable cannabinoid particles, patients could avoid side effects such as liver damage, due to lower dosing, while achieving better control of effects (Eisenberg et al., 2014; Almog et al., 2020).

## 4 Pharmacology and beneficial polypharmacy “made by nature”

### 4.1 The role of cannabinoid polypharmacology

Cannabinoids have many targets from a broad range of protein families—which can be seen as disadvantage as the role of an individual target is hard to decipher, or as an advantage in the sense of beneficial polypharmacology. Drugs showing polypharmacology instead of being highly selective have proven useful in various examples (Roth et al., 2004; Anighoro et al., 2014). Examples are COX inhibitors such as non-steroidal anti-inflammatory drugs (NSAID) (Brune and Patrignani, 2015). Interestingly, parts of the mechanism of action (paracetamol) are due to interactions with the endocannabinoid system. AM404, the metabolite of paracetamol, interacts with CB1 receptors (Deshpande and DeLorenzo, 2011). This interaction is believed to drive the anticonvulsant effect of paracetamol. Furthermore, AM404 targets TRPV1 and inhibits COX (Zygmunt et al., 2000). However, the mode of action on thermoregulation and analgesia is not fully understood. Nevertheless, paracetamol is one of the most used compounds globally and a good example of a polypharmacological compound with ECS interactions.

Especially, because plant compounds such as cannabinoids (Izzo et al., 2009; Ibeas Bih et al., 2015) or others (Barrajón-Catalán et al., 2014; Sharma et al., 2016; Kong et al., 2021) display polypharmacology rather than being highly selective compounds, they are of great interest in complex and multifaceted disorders, such as chronic pain or epilepsies. Polypharmacology offers the advantage that a single prescription (or a single compound) could cover comorbid indications, such as e.g., spasticity and insomnia, reducing the need for multiple medications and thus also reducing side effect burden for patients with multiple conditions (Nutt et al., 2022). These considerations are currently not reflected in first-line treatment guidelines. Most of the active ingredients of cannabis based medications show polypharmacology (Haller et al., 2020, 2010; Russo, 2017; Sulak et al., 2017; Bonn-Miller et al., 2018; Lewis et al., 2018; Nahler and Jones, 2018).

It is noteworthy in this context that the use of medical cannabis led to the reduction of prescription drugs, including opioids, non-opioids, antidepressants, anti-seizure drugs and benzodiazepines. Furthermore, an improvement of overall quality of life in Canadian cannabis patients was observed (Lucas et al., 2021). These findings are not limited to medical cannabis, because cannabinoid medications like Sativex^®^ may also reduce opioid use (Bramness et al., 2022).

Several concepts have been developed to understand and evaluate the potential of botanicals, especially complex extracts containing cannabinoids in complex disease. Synergistic effects exerted by way of polypharmacology have been termed “pharmacological handshake” (Brodie et al., 2015).

### 4.2 Polypharmacy meets polypharmacology

To raise the complexity of the matter, chemovars of the same plant species show distinct effects, even if their main active ingredients are standardized. The remaining plant matrix contains numerous other active molecules and those vary between chemovars. More than thousand chemovars of *Cannabis sativa* have been identified, and a growing number is used by doctors and patients. Synergistic effects from multiple cannabis constituents are often referred to as the “entourage effect”. Historically, this term derived from early conducted experiments investigating endocannabinoid function (Ben-Shabat et al., 1998; Anand et al., 2021).

Synergistic effects of cannabinoids have not only been seen in cannabis preparations. For instance alkamides, derived from *Echinacea*, act *via* CB1 and CB2 receptors (Gertsch et al., 2006; Haller et al., 2010; Hohmann et al., 2011), and elicit diverse effects including immunomodulatory, antidepressant and anxiolytic effects. Especially the anxiolytic effects are displayed only by certain arrangements of distinct alkamides (Haller et al., 2020). These are compounded and marketed as EP107™ in several supplements such as AnxioControl^®^ (Hungary), AnxioCalm^®^ (United States and Canada), AnxioFree^®^ (Slovenia), HERBALANX^®^ (Turkye) and cannanx^®^ (Germany and Austria).

Thus, multiple constituents of a plant have been shown to act together in the sense of a “beneficial polypharmacy”, and this is the case also for the currently used defined extracts that combine CBD, Δ9-THC, and other chemicals in a beneficial defined mixture. Thus, cannabis-based extracts consisting of multiple constituents display polypharmacy of a collection of compounds, with each contributing its own specific polypharmacology. This poses big challenges for the understanding of mechanisms of actions, but likely is highly beneficial (Brodie et al., 2015).

## 5 Emerging roles of Cys-loop receptors in the target space of cannabinoid pharmacology

Here we briefly review current knowledge about the roles of Cys-loop receptors in the pathobiology or pharmacology of the clinical entities in which cannabinoids have been demonstrated effectiveness - chronic pain, epilepsies and selected further diseases of the nervous system based on the recent findings on cannabinoid clinical effects (Table 3 and 4).

### 5.1 Epilepsy and seizures

Epilepsy has been understood as a spectrum of neurological disorders, with ongoing prevalence of episodes containing epileptic seizures, affecting approximately 65 million people globally. Several forms of epilepsy comprise individual diagnostic entities, and multiple causes such as structural changes, genetic disposition (>500 genes identified), infectious disease, metabolic influence, autoimmune disorders and other unknown aetiologies, have been identified. More than 20 drugs have been approved by regulators to treat epilepsy but barely two-third achieve seizure control by first-line treatment anti-seizure drugs (ASD). However, first-line drugs are often unfavorable to disease progression, and misdiagnosis leads to inappropriate prescriptions further severely affecting patients. Novel treatment options, combined with faster and more precise diagnosis, is expected to result in updated guidelines for first-line drugs (Wirrell et al., 2017; Devinsky et al., 2018a).

#### 5.1.1 Cannabinoids in clinical use against epilepsies

CBD is successfully used as a registered drug (Epidiolex^®^) for severe and rare forms of epilepsy in children (Dravet-syndrome (DS), Lennox Gastaut Syndrome (LGS) and Tuberous Sclerosis Complex (TSC). In tested TSC patients, 89% was taking ≥2 AED’s (anti epileptic drugs) and still experiencing a mean of 57 TSC-associated seizures per 28 days. The most commonly used concomitant AEDs were valproate (45%), vigabatrin (33%), levetiracetam (29%), and clobazam (27%). The associated seizures were reduced 2x by Epidiolex^®^ compared to placebo in a 16-week clinical trial (Devinsky et al., 2016; Thiele et al., 2018; Thiele et al., 2021; Wu et al., 2022; Thiele et al., 2022, Jazz Pharmaceuticals, 2022). In tested LGS patients, 94% of patients were taking ≥2 AEDs at baseline and still experiencing a median of 74 and 85 drop seizures (Thiele et al., 2018; Devinsky et al., 2018b) respectively, per 28 days. The most frequently used concomitant AEDs across Thiele et al., 2018; and Devinsky et al., 2018a; were clobazam (49%), valproate (39%), lamotrigine (33%). Epidiolex^®^ significantly reduced seizure frequency (2.5x compared to placebo) in highly refractory patients with LGS (Devinsky et al., 2018b; Thiele et al., 2018; Patel et al., 2021; Privitera et al., 2021; Jazz Pharmaceuticals, 2022). Epidiolex^®^ significantly reduced convulsive seizures (3x compared to placebo) in patients with Dravet syndrome. Patients at baseline had previously tried a median of 4 prior AEDs and are currently uncontrolled with a median of 3 current AEDs. The most frequently used concomitant AEDs were clobazam (65%), valproate (57%), stiripentol (43%); 93% of patients were taking ≥2 AEDs at baseline and still experiencing a median of 13 convulsive seizures per 28 days (Devinsky et al., 2017; Scheffer et al., 2021; Jazz Pharmaceuticals, 2022). Additional types of epilepsy have been investigated as indicated by Bilbao and Spanagel, 2022 in clinical trials with promising results.

#### 5.1.2 Genetic, postmortem, and preclinical findings

Genetic epilepsy syndromes, often caused by a variation in a single epilepsy-associated risk gene, affect up to 50% of epilepsy patients (Hernandez and Macdonald, 2019) and have major implications in treatment failures with first-line treatment ASDs (Ahring et al., 2022a).

However, most idiopathic generalized epilepsies (IGE) are thought to have a genetic component, and it is becoming increasingly clear that mutations of transmembrane ion channels, both voltage-gated and ligand-gated, are the cause of many forms of human epilepsies. Mutations in Cys-loop receptors belong to one of the best studied causes of epilepsy. In the case of GABAA receptors, monogenic GABR mutations are clustered according to their pathomechanism which can either affect channel transcription or translation, trafficking, folding, truncation, ER retention, negative effect on other subunits or lack of endogenous modulation (Hernandez and Macdonald, 2019; Vogel et al., 2022). It has been proposed that among paralog subunits, variations in the same structural section of the protein potentially cause similar functional deficiencies due to the high homology. More than 60 *de novo* mutations were found in patients with varying forms of epileptic encephalopathies (EE) and it seems that especially mutational burden at conserved regions within the transmembrane domain (TMD), such as the pore forming TM2 segment, are hot spots for severe forms of EE.

So far, mutations in GABAAR subunits α1, β2, β3, γ2, δ and ε have been associated with GESs (Dibbens et al., 2009; Kang and Macdonald, 2009; Johannesen et al., 2016; Hamdan et al., 2017; Møller et al., 2017; Markus et al., 2020; Ahring et al., 2022a) but more mutated GABR genes were found in patients with epilepsy, such as α2, α4, α5 and β1 (Allen et al., 2013; Lien et al., 2016; Butler et al., 2018; May et al., 2018; Vogel et al., 2022). The subunits encoded by GABR genes found relevant in epilepsies contribute to receptors which mediate both phasic and/or tonic inhibition (Guazzi and Striano, 2019). Change of GABAAR function impacts on the balance of excitation and inhibition, with downstream disinhibition and hyperexcitability of neurons. Interestingly, not only molecular loss of function, but also molecular gain of function and complex change of function phenotypes have been connected with seizure disorders (Ahring et al., 2022a, 2022b; Absalom et al., 2022; Vogel et al., 2022). Many GABR variants have been compiled and reviewed recently (Fu et al., 2022), and an overview is provided in Supplementary Table S2.

Interestingly, the first gene that was associated with monogenic epilepsy in 1995, the ChRNA4, encoding the nicotinic acetylcholine receptor α4 subunit, was found in a patient with a mutation in the TMD and a similar change of channel desensitization as found for GABAA receptor mutations at homologous positions. This is suggestive of further structural-mutational correlation within the Cys-loop family. For nAChRs, involvement in epilepsy is now firmly established. Mutations in the TM2 and TM3 domains of α4 and β2 subunits are associated with sleep-related hypermotor epilepsy (SHE), formerly known as autosomal dominant nocturnal frontal lobe epilepsy (Steinlein, 2014; Becchetti et al., 2020; Fukuyama et al., 2020; Weltzin et al., 2021). Furthermore, α7-nAChR subunits are expressed throughout the brain with a majority in hippocampus and cortex and have been shown to be an essential regulator of seizure susceptibility. A decrease in the activity of α7-nAChRs could increase the excitability of CA1 pyramidal neurons in the hippocampus. Furthermore, reduced α7-nAChR expression in human epileptogenic tissues has been found, and indicates a risk for seizure susceptibility. (Banerjee et al., 2020; Sun et al., 2021).

Not only for nAChRs, but also for specific GABR gene products, post-mortem changes have been observed. GABAAR-α4 subunit mRNA and protein concentration was increased in hippocampal specimens of drug resistant temporal lobe epilepsy (TLE) patients who underwent epilepsy surgery (Sperk et al., 2021). The GABAAR α4 subunit concentration in epileptic tissue was three times higher than compared to control tissue across multiple pathologies such as focal cortical dysplasia (FCD), tuberous sclerosis complex (TSC), ganglioglioma, and gliosis, suggesting enhanced α4 subunit expression may occur due to recurrent seizures rather than epilepsy etiology (Joshi et al., 2020).

Furthermore, RT-PCR revealed a significant difference in the expression of GABAAR α1, α2, and α4 subunits, whereas the expression of α1 subunit mRNA was significantly lower and the expression of α2 and α4 subunits were significantly higher in Dravet syndrome patients compared to controls (Ruffolo et al., 2018). Preclinical research further substantiates both causative and compensatory roles of changes in expression levels (citations, e.g., in multiple animal models of epilepsy (Brooks-Kayal et al., 1998; Sperk, 2007; Sperk et al., 2021).

All *in vitro* and preclinical evidence suggests that CBD enhances GABA effects in a majority of the tested receptor subtypes (Bakas et al., 2017; Schmiedhofer 2017), similarly to other GABAAR targeting antiepileptic medications such as benzodiazepines or barbiturates with ATC codes as antiepileptic. Thus, it is very plausible that the GABA-ergic effects contribute to the antiepileptic effects of CBD, potentially as part of a beneficial target portfolio that might also include effects on other ionotropic receptor species. Moreover, GABAARs play pivotal roles in the pathophysiology of epilepsy syndromes.

### 5.2 Chronic pain

Chronic pain affects 20% of the population and is classified into different categories. These are chronic primary pain, chronic neuropathic pain, chronic cancer-related pain, chronic posttraumatic pain, chronic secondary headache, chronic visceral pain, and chronic secondary musculoskeletal pain according to ICD11 (International Classification of Diseases). As a result of chronic pain, biopsychosocial factors like a patient’s behavioral and emotional characteristics, socioeconomic factors, and overall quality of life, require a more complex treatment spectrum than acute pain management. Further considerations regarding distinct clinical evaluations and guidelines are necessary (Urits et al., 2020). Both inflammation and neuropathy are major sources of chronic pain and hyperalgesia (Zeilhofer et al., 2021). Chronic pain is often treated with opioids.

#### 5.2.1 Cannabinoids in clinical use against chronic pain

Cannabinoids have shown to be of relevance in the treatment of chronic pain (Johnson et al., 2010, 2013; Portenoy et al., 2012; Johal et al., 2020; Bilbao and Spanagel, 2022; Henson et al., 2022).

Δ9-THC as a single compound has been shown to be effective in chronic pain (Weizman et al., 2018; van Amerongen et al., 2018) but superior than other drugs (Rintala et al., 2010) considering pain control as the only primary endpoint. Furthermore, using synthetic cannabinoids as add-on therapy (Pinsger et al., 2006; Wissel et al., 2006) or alone (Karst et al., 2003) has been shown to be effective in chronic pain.

The clinical use of cannabinoid preparations such as Sativex^®^, containing Δ9-THC and CBD has been shown to be successful in the alleviation of chronic pain (Bilbao and Spanagel, 2022). Despite pitfalls in reaching primary endpoints in clinical investigation (Berman et al., 2004; Serpell et al., 2014; Lichtman et al., 2018), secondary endpoints were reached improving the overall quality of life of patients. Furthermore, both compounds display individual and overlapping routes of action in pain alleviation, studied respectively (Russo, 2008). Furthermore, pain alleviation is potentiated when CBD is added to Δ9-THC preparations (Johnson et al., 2010). Cannabinoid use (CBD/Δ9-THC, Δ9-THC and CBD) in chronic neuropathic pain is associated with better “safety-benefit-score” compared to Duloxetine, gabapentinoids, amitriptyline, tramadol, ibuprofen, methadone, oxycodone, morphine and fentanyl (Nutt et al., 2022). However, adding CBD to Δ9-THC treatments minimizes side effects of Δ9-THC and adds polymodal effects in clinical use (Martínez-Aguirre et al., 2020; Melas et al., 2021; Henson et al., 2022). Inhalation of smoked cannabis (25 mg Δ9-THC) reduced the intensity of neuropathic pain, improved sleep and was well tolerated (Ware et al., 2010) while pure Δ9-THC, taken orally at this dosage, would confer a risk of potential side effects (Kraft et al., 2008; Rintala et al., 2010).

Add-on therapies of cannabinoid preparations are possible during opioid treatment (Haroutiunian et al., 2008) and led to a reduction of opioid use proposing safer long term treatments (Abrams et al., 2011; Capano et al., 2020; Lucas et al., 2021; Bramness et al., 2022).

Interestingly, there is evidence that cannabinoids can display sex dependent effects (Bassir Nia et al., 2022). Therefore, sex differences must be taken into account in study design. However, because controversial evidence exists (Arkell et al., 2022), in some studies women were excluded regarding fluctuations of pain sensitivity over menstrual cycle (Weizman et al., 2018), and others are done only on women, not addressing the underlying mechanisms of sex dependent effects at all (Kraft et al., 2008).

In summary, there is little qualified information available to define the exact effectiveness of cannabinoids in chronic pain in general, but the effectiveness probably outweighs the side effects (Mücke et al., 2018). Therefore concepts such as the “pharmacological handshake” seem profoundly logical in certain medical conditions to quantify the clinical purpose of polypharmacological use (Brodie et al., 2015). To test such “handshake-models” for synergistic effects on comorbid medical conditions, or a range of symptoms (such as inflammation and pain), multiple outcomes need to be evaluated and integrated “net outcomes” used to compare different drugs, or drugs with placebo.

#### 5.2.2 The involvement of Cys-loop receptors in chronic pain

Pharmacologically enhanced inhibition mediated by GABAARs or GlyRs in the spinal cord has been shown to reverse hyperalgesia and allodynia in various pain models. The underlying mechanism of inhibitory impairments in the development and progression of neuropathic pain is far from being fully understood. However, both GABAARs and GlyRs are intensely investigated as potential targets for the alleviation of chronic pain (Gradwell et al., 2020; Zeilhofer et al., 2021).

Modulators of extrasynaptic GABAA receptors preferentially targeting α4βδ containing GABAA receptors, such as allopregnanolone, have been shown to alleviate chronic and neuropathic pain in animals and humans (Afrazi and Esmaeili-Mahani, 2014; Fujita et al., 2018; Aoyama et al., 2019; Luo et al., 2021).

α5-containing GABAARs are expressed in laminae I-II of the spinal cord neurons, sensory neurons, and motor neurons. It is suspected that α5-containing GABAARs do not modulate acute nociception. However, they may play a pro-nociceptive role at late stages of inflammation and are putative targets for chronic and neuropathic pain. Blocking of α5-containing GABAARs has been found to reduce chronic pain in animal models (Delgado-Lezama et al., 2021). Interestingly, sex-dependent effects have been observed in the reduction of stress-induced behaviors, and in chronic pain, both linked to α5-containing GABAARs modulation. Both, α5-containing GABAARs mRNA and protein changes in DRG and spinal cord, are modulated in a sex-dependent manner. Furthermore, nerve injury increases the expression of α5-containing GABAARs in female DRG and spinal cord in female, but not in male rodents. Distinct DNA methylation and hormonal adaptations are associated with these observations. These results suggest the α5-containing GABAARs putative targets of chronic pain in females (Delgado-Lezama et al., 2021; Franco-Enzástiga et al., 2021).

A mechanistically novel treatment option for trigeminal neuropathic pain has been suggested recently. A PAM of α6-containing GABAARs alleviated and prevented trigeminal neuropathic pain in a rat lesion model (Vasović et al., 2019; Sieghart et al., 2022). Furthermore, α6-containing GABAAR-selective PAMs are potential anti-migraine agents (Tzeng et al., 2021). These effects are thought to be linked to α6-subunit containing receptors in the trigeminal ganglion (Sieghart et al., 2022). This is interesting since Δ9-THC and CBD have shown strong positive modulation in α6βδ containing receptors. However, both compounds have been identified as positive allosteric modulators of various GABAA receptors (i.e., are largely unselective agents). These findings thus may support the hypothesis of involvement of GABAA receptors in pain reduction due to enhanced tonic and synaptic inhibition (Bakas et al., 2017; Schmiedhofer, 2017).

Novel and highly selective GlyR potentiators represent a novel class of compounds. Specific glycinergic interneuron modulators have been identified and their effects serve as preclinical proof of concept for the activation of glycinergic circuitry as a strategy for treating neuropathic pain (Huang et al., 2017).

The role of GlyR-α3 in inflammatory pain and its identified implications in pain modulation had been supported by the analgesic effects of Δ9-THC and its derivatives (Xiong et al., 2011). GlyR-α3 is highly enriched in the superficial layers of the spinal dorsal horn, a key site of nociceptive processing (Zeilhofer et al., 2021).

Furthermore, both CBD and DH-CBD target α3-GlyR. DH-CBD is a modified form of CBD with higher efficacy on GlyR and showing greater effects in chronic pain models (Xiong et al., 2012). α3-GlyR subunits are thought to be essential for the cannabinoid induced analgesic effect in chronic pain, based on observations in GlyR α3^−/−^ knock-out mice (Xiong et al., 2012). In addition, a point-mutation of GlyR-α1S296A significantly inhibits DH-CBD potentiation of glycine currents in HEK-293 cells and neurons of spinal cord slices. Intriguingly, the DH-CBD-induced reduction of analgesia for inflammatory pain was absent in GlyR-α1S296A knock-in mice, suggesting that spinal α1-GlyRs are also potential targets for cannabinoid analgesia in chronic inflammatory pain (Lu et al., 2018).

Thus, preclinical evidence for the involvement of specific GlyR and GABAAR assemblies in different aspects of chronic pain formation, maintenance and treatment is established and supportive of therapeutic benefits by cannabinoids targeting these receptors. The example of DH-CBD shows how modified molecules can be used to specifically target existing cannabinoid targets.

Polypharmacology involving the eCB system and exogenous cannabinoids may play a pivotal role in pain and inflammatory processes. For instance, NA-Gly, a metabolite of AEA, which is produced on demand after phases of high neuronal excitation, shows distinct pharmacological effects on Cys-loop receptors (GABAR and GlyR), depending on subunits, compared to AEA. However, synergistic effects could be achieved by direct interaction with endogenous molecules, especially with compounds of lipid signaling pathways. Interestingly, arachidonic acid the precursor molecule of AEA, as well as other acidic cannabinoids such as NA-Gly, has been shown to inhibit GlyR-α2/α3 and potentiate GlyR-α1, while neutral cannabinoids such as AEA potentiates all of them. Since subunit expression can vary during inflammation, a better understanding of endogenous cannabinoid pharmacology on Cys-loop receptors is needed, to establish the underlying physiological mechanism of inflammatory regulation. However, it is possible that transitions in endogenous GlyR response act as possible regulation during inflammation not only in neuropathic or chronic pain. This possibly suggests how the therapeutic setting is related to disease progression and how it directly influences the effects on the molecular level.

Furthermore the direct influence of COX2 and further PGE2, as a inhibitor of GlyR-α3, on the development of chronic and neuropathic pain, during inflammation has been proposed, but is still controversial (Ma et al., 2010; Zeilhofer et al., 2021). Interestingly, phytocannabinoids have been shown to have inhibitory effects on COX2 activity and may contribute in a polypharmacological manner to the alleviation of chronic and neuropathic pain (Ruhaak et al., 2011). The absence of potential harmful side effects of GlyR modulators, such as muscle relaxation, respiratory dysfunction and addiction, raises hope for future treatment options (Zeilhofer et al., 2021).

### 5.3 Other disorders of the nervous system

In the meta-review by Bilbao an Spanagel, (2022) high and moderate effectiveness of phytocannabinoids was seen in several indications with established Cys-loop receptor involvement in either the pathobiology, or as therapeutic targets.

Many sleeping aids (hypnotics) act by PAM effects on GABAARs, such as all benzodiazepines (Sigel and Ernst, 2018). While other targets can also elicit hypnotic effects, it seems plausible that the overall effectiveness of Δ9-THC might be at least in part mediated also by its PAM effects on a range of GABAA receptors.

Tic disorders including Tourette syndrome are also under investigation from the viewpoint of GABAAR pathobiology and therapeutic usefulness. Tourette syndrome has been shown to present with widespread abnormality of the γ-aminobutyric acid-ergic system (Lerner et al., 2012). In mouse models of Tic disorders, an α6-preferring PAM was found to rescue the sensorimotor gating deficits, hinting at potential therapeutic use of compounds which act on cerebellar, α6 containing GABAA receptors, reviewed in (Sieghart et al., 2022).

For different symptoms of psychotic disorders, ongoing clinical trials and the literature suggest possible promise for pCBs (see Table 4). Among the Cys-loop receptors, specific populations of GABAARs and nAChRs have been investigated for putative pathobiological and therapeutic roles in psychotic disorders.

α5-containing GABAARs are believed to mediate tonic inhibition in hippocampal CA3 and CA1 pyramidal neurons, cortical neurons and contribute to tonic inhibition in dentate gyrus granule cells. PAM of α5-containing GABAARs are proposed potential therapeutics for some neurodevelopmental disorders, depression, schizophrenia, and mild cognitive impairment (Jacob, 2019). Preclinical evidence suggests that blockade of α5-containing GABAARs leads to behavioral phenotypes associated with schizophrenia. Furthermore, postmortem evidence indicates lower hippocampal α5-containing GABAARs protein and mRNA levels in schizophrenia (Marques et al., 2021). Adding that NAM effects reduce chronic pain implies that compounds with high efficacy for either NAM or PAM effects on α5-containing GABAARs could have negative side effects on each group of indications. In addition, α6-containing GABAA receptor-selective PAMs (chiefly found in the cerebellum) are potential medicines for treating sensorimotor gating deficits in neuropsychiatric disorders (Chiou et al., 2018), thus offering a possible alternative to compounds with primary effects on α5-containing GABAARs.

A reduction of α7-AChR has been reported in Alzheimer’s and schizophrenic patients. CHRNA7 is genetically linked to multiple disorders with cognitive deficits, including schizophrenia, intellectual disability, bipolar disorder, autism spectrum disorders, attention deficit hyperactivity disorder, epilepsies, Alzheimer’s disease, and sensory processing deficits (Corradi and Bouzat, 2016).

Furthermore, there is now extensive preclinical evidence that α7-nAChR ligands have pro-cognitive effects and other properties that are considered to be beneficial to schizophrenia patients. The development of nAChR modulators have been considered as an effective therapeutic strategy to improving psychiatric symptoms and e.g. promoting smoking cessation in schizophrenia patients. However, like the other pro-cognitive strategies, no α7-nAChR ligand has been approved for clinical use in schizophrenia thus far (Parikh et al., 2016; Terry and Callahan, 2020). Substance abuse disorders, including but not limited to nicotine, also are an emerging indication for cannabinoid medications, and involve Cys-loop receptors in the genetic and pathobiological components of SUDs.

As far as further indications are concerned, limited evidence was presented in clinical data by (Bilbao an Spanagel, 2022), but mechanistic properties and analogous pharmacological approaches suggest a possible promise for cannabinoids in mood disorders such as premenstrual dysphoric disorder (PMDD).

A lack in tonic inhibition could trigger the dysphoric symptoms and states of anxiousness during PPD and PMD due to reduced expression of extrasynaptic subunits of GABAA receptors (Hantsoo and Epperson, 2020). Furthermore, neurosteroid alterations in different situations as shown in postpartum depression (PPD) postmenarche or during menstruation cycle have direct impact on the expression of GABAAR subunits, α4 subunits in particular (Smith et al., 2009; Andrade et al., 2012; Modgil et al., 2017). However, extrasynaptic receptors are highly affected endogenously by neurosteroids and therapeutic approaches with brexanolone, a PAM of extrasynaptic receptors, and other synthetic allopregnanolone analogs are in clinical phases. Thus, new therapeutics are welcome to specifically target extrasynaptic GABAA receptors because tonic inhibition is modulating the gain and tone of these neuronal systems (Semyanov et al., 2004). Non-hormonal alternative PAMs of extrasynaptic GABAA receptors based on pCBs (Bakas et al., 2017; Schmiedhofer, 2017) deserve serious consideration Furthermore, polypharmacological compounds like CBD could address multicausal etymology and PMDD symptoms such as anxiety due multiple targets, e.g. the 5-HT1A receptor agonism.

## 6 Outlook

Phytocannabinoids (plant based cannabinoids) are widely used for recreational and medicinal purposes, which in turn leads to basic scientific research casting more light on the role and involvement of the ECS and expanding the understanding of the functions of the “endocannabinoidome” in human physiology. At this point, various molecular interaction sites for cannabinoids emerge, encompassing different targets in addition to the classical CB receptors.

In most countries cannabis medication is the last resort treatment option. This is not in line with the emerging clinical evidence of superiority of cannabinoids in medicine. In the coming years a wealth of new clinical data in cannabinoid research will most likely indicate advantages of whole plant preparations over single compounds. This refers to synergistic effects of plant compounds not limited to cannabinoids, but also to terpenoids and flavonoids and other compounds, which in turn will expand the reservoir for clinical investigation but also complicate the design of trials, methods, and proof of concepts.

Therefore, models such as the “pharmacological handshake/polypharmacological portfolio” will gain interest, especially in economical harsh times accompanied by targeted saving measures, in countries with access to recreational and medicinal cannabis. War times, and post COVID, will lead to differing measures, as anxiety disorders, sleep disorders and PTSD will rise dramatically.

Future indications of cannabinoids, as reflected by preclinical evidence, clinical trials, literature, and off-label use, include neuropsychiatric indications, specifically anxiety disorders incl. PTSD; mood disorders including depression and premenstrual dysphoric disorder (PMDD); substance abuse disorders (SUDs); psychotic disorders incl. schizophrenia; sleep related disorders and stress related conditions. In addition, non-neuronal conditions including cancer, inflammatory and immune system mediated disorders such as Crohn’s disease, IBD (inflammatory bowels disease) and colitis ulcerosa; metabolic disorders such as diabetes and metabolic syndrome; emergency states such as stroke and neonatal ischemia also appear highly promising.

It is intriguing to note the big overlap between the outcome-based list of putative neuropsychiatric indications for cannabis based medications, and the pathways that connect Cys-loop receptors with neuropsychiatric function: Anxiety disorders, sleep related disorders and seizure disorders are classically treated with GABA-ergic medications, and cannabinoids act as GABA-ergics. The role of different GABAAR subtypes in cannabinoid influence on sleep/wake states, on anxiety, and on seizure threshold is not understood and offers many avenues of investigation. Importantly, the effects of several cannabinoids have been compared between CB1/CB2−/− knock-out (KO) mice and GABAA-β2−/− KO mice respectively. While both 2-AG and NE have shown strong hypomotility in WT and CB1/CB2−/− KO mice, hypermotility was observed in GABAA-β2−/− KO mice. These findings suggest a cannabinoid receptor independent, sedative effect of the endocannabinoids 2-AG and noladin ether (NE), most likely conducted *via* an involvement of GABAA-β2 subunit containing receptor function (Sigel et al., 2011). To further disentangle the physiological interplay between Cys-loop receptors and cannabinoid receptors, there is a need for further *in vivo* experiments, using various KO-animals treated with different cannabinoids (and their blocking agents if available) respectively. However, bearing in mind that physiological levels of endocannabinoids and other endogenous molecules, such as neurosteroids, complicate the interpretation of effects, testing of exogenous ligands seems advisable.

For other Cys-loop receptor families, similar arguments apply: The antiemetic actions of cannabis based medications are in line with the molecular effects on 5HT3Rs, beneficial influence on SUDs might be connected to actions on nACh and GABAA receptors. Furthermore, non-neuronal Cys-loop receptors found in immune cells, in cancer cells and other peripheral cell types might contribute to other beneficial effects (Tables 3, 4, Bilbao and Spanagel, 2022). More complete insight into the effects of cannabinoids on Cys-loop receptors is still required to postulate putative links for neuronal and non-neuronal receptor subtypes with therapeutic effects, as evidenced by the many gaps and low numbers of investigated family members in Figure 3. Since pCBs broadly target Cys-loop receptors, beneficial polypharmacology can potentially already occur due to this single target family—e.g., in beneficial influences on sleep and anxiety *via* GABAARs, on pain *via* Gly and GABAARs together with antiemetic and appetite stimulating effects *via* 5HT3Rs in cancer patients undergoing chemotherapy.

## Data Availability

Supporting data is available in the Supplementary Material, and further inquiries can be directed to the corresponding authors.

## Abbreviations

2-AG, 2-arachidonoylglycerol; AEA, anandamide, arachidonoylethanolamine; API, active pharmaceutical ingredient; CA1/CA3, cornu ammonis; CB, cannabinoid; CBD, cannabidiol; CBG, cannabigerol; COX2, cyclooxygenase; DRG, dorsal root ganglion; eCB, endocannabinoid; GABA, gamma amino butyric acid; Gly, glycine; GlyR, glycine receptor; GPR, G-protein coupled receptor; HEK253, human embryonic kidney cells; mRNA, messenger ribonucleic acid; NAM, negative allosteric modulation; NSAID, non-steroidal anti-inflammatory drugs; PAM, positive allosteric modulation; pCB, phytocannabinoid; PMDD, premenstrual dysphoric disorder; PMS, premenstrual syndrome; PTSD, posttraumatic stress disorder; SAM, silent allosteric modulation; sCB, synthetic cannabinoid; SSRI, selective serotonin reuptake inhibitor; THDOC, 3α, 21-dihydroxy-5α pregnan-20-one; TMD, transmembrane Domain; Δ9-THC, tetrahydrocannabinol.
