# Supplementary material for: Cys-loop receptors on cannabinoids: All high?
Source: Front Physiol. 2022 Nov 9;13:1044575. doi: 10.3389/fphys.2022.1044575 (PMC9682269; doi:10.3389/fphys.2022.1044575)
Supplement: Supplementary file 1 [file DataSheet1.PDF]

Supplementary Material

1 Supplementary Data

Table 1 Cannabinoid effects on cys-loop receptors:

Anandamide (AEA)
Arachidonylethanolamide

Receptor	Effects	Results	max. potentiation / max. inhibition	System	Source
GlyR α1	PAM	EC50 319 nM ±31 nM		X. laevis oocytes	(Hejazi et al. 2006)
GlyR α1β1	PAM	EC50 318 nM ±24 nM		X. laevis oocytes	(Hejazi et al. 2006)
GlyRα1	PAM	EC50 38 nM ±11 nM		HEK293 cells	(Yang et al. 2008)
GlyRα1β	PAM	EC50 75 nM ± 20 nM	128 ±33%	HEK293 cells	(Yang et al. 2008)
GlyR α2		-		HEK293 cells	(Yang et al. 2008)
GlyR α3		-		HEK293 cells	(Yang et al. 2008)
GlyR α1	PAM		~160 %*	HEK293 cells	(Yévenes and Zeilhofer 2011)
GlyR α2	PAM		~90 %*	HEK293 cells	(Yévenes and Zeilhofer 2011)
GlyR α3	PAM		~110 %*	HEK293 cells	(Yévenes and Zeilhofer 2011)
GlyR	PAM		I/ICTRL(*100%) 20 ±7% at 1 µM	hippocampal CA1 and CA3 pyramidal neurons and Purkinje neurons	(Lozovaya et al. 2005)
GlyR α1	PAM				
GlyR α1	PAM	EC50 Glycine + AEA: 5.5 ± 2.0 µM	86 ± 24 % at 1 µM AEA 800 ± 71% at 30 µM AEA	spinal neurons	(Xiong et al. 2012)
GlyR α1 / GlyR α1β	PAM	EC50 Glycine + AEA: 4.2 ± 1.95 µM	97 ± 16% / 85 ± 12% at 1 µM AEA	HEK293 cells	(Xiong et al. 2012)
GABAAR α2β3γ2		-		X. laevis oocytes	(Hejazi et al. 2006)
GABAAR α1β2γ2	PAM	minor PAM effects at 1 µM and 3 µM		X. laevis oocytes	(Sigel et al. 2011)

5HT3A	NAM	inhibition		X. laevis oocytes,<br>HEK293 cells	(Xiong et al. 2008)
5HT3A	NAM	IC50 129.6 nM		HEK293 cells	(Barann et al. 2002)
5HT3A		1 µM 46%<br>10 µM 41%<br>100 µM 29%		binding assay	(Kimura et al. 1998)
nAChR α4β2	NAM	IC50 (at 20 min) of approximately 300 nM		SH-EP1 cells	(Spivak et al. 2007)
nAChR α4β2	NAM	IC50 value of 0.9 ± 2 µM		thalamic synaptosomes	(Butt et al. 2008)
nAChR α7	NAM	10 nM to 30 µM<br>IC50 229.7nM ±20.4 nM		X. laevis oocytes	(Oz et al. 2003)

2-Arachidonoylglycerol (2-AG)

Receptor	Effects	Results	max. potentiation / max. inhibition	System	Source
GABAAR α1β2γ2	PAM	EC50 2.1 ± 0.5 µM<br><br>subunit selectivity β2 > β3 (1/3) > β1 (no effect)<br><br>superadditivity between THDOC and 2-AG.	138 ± 21%	X. laevis oocytes	(Sigel et al. 2011)
GABAAR α1β2δ	PAM	EC50 2.9 ± 1.8 µM		X. laevis oocytes	(Sigel et al. 2011)
GABAAR α1β2γ2	PAM	binding site at M3-M4 interface of β2 subunit		X. laevis oocytes<br>Homology modeling and docking<br>site-directed mutagenesis	(Baur et al. 2013)
GABAAR α1β2γ2L	PAM	EC50 GABA: 181.3 µM (127.0 – 258.7)<br>EC50 GABA + 10 µM 2-AG: 2.4 µM (0.8 – 6.7)	88.9 % (70.6 – 99.4)	X. laevis oocytes	(Bakas et al. 2017)
GABAAR α2β2γ2L	PAM	EC50 GABA: 214.5 µM (157.7 – 255.3)<br>EC50 GABA + 10 µM 2-AG: 15.7 µM (8.2 – 30.0)	290.6 % (223.5 – 357.7)	X. laevis oocytes	(Bakas et al. 2017)
GABAAR α2β2(V436T)γ2L	PAM	EC50 GABA: 219.2 µM (185.5 – 259.0)<br>EC50 GABA + 10 µM 2-AG: 6.0 µM (1.4 – 11.9)	99.1 % (74.1 – 124)	X. laevis oocytes	(Bakas et al. 2017)
GABAAR α3β2γ2L	PAM	EC50 GABA: 155.1 µM (96.1 – 250.6)<br>EC50 GABA + 10 µM 2-AG: 14.7 µM (6.8 – 31.3)	127.0 % (93.0 – 161.0)	X. laevis oocytes	(Bakas et al. 2017)
GABAAR α4β2γ2L	PAM	EC50 GABA: 84.6 µM (62.7 – 114.1)<br>EC50 GABA + 10 µM 2-AG: 3.9 µM (2.1 – 7.3)	114.7 % (95.3 – 134.1)	X. laevis oocytes	(Bakas et al. 2017)
GABAAR α5β2γ2L	PAM	EC50 GABA: 24.2 µM (21.4 – 27.3)<br>EC50 GABA + 10 µM 2-AG: 1.5 µM (0.3 – 8.2)	98.3 % (64.1 – 132.5)	X. laevis oocytes	(Bakas et al. 2017)

GABAAR α6β2γ2L	PAM	EC50 GABA: 13.4 μM (7.5 – 24.3)<br>EC50 GABA + 10 μM 2-AG: 6.4 μM (1.2 – 35.5)	118.6 % (62.8 – 174.4)	X. laevis oocytes	(Bakas et al. 2017)
GABAAR α2β1γ2L	PAM	EC50 GABA + 10 μM 2-AG:13.3 μM (8.2 – 21.3)	142.1 % (113.2 – 169.4)	X. laevis oocytes	(Bakas et al. 2017)
GABAAR α2β3γ2L	PAM	EC50 GABA + 10 μM 2-AG: 9.8 μM (6.5 – 14.6)	239.7 % (204.4 – 275.1))	X. laevis oocytes	(Bakas et al. 2017)
GABAAR α4β2δ	PAM	EC50 GABA + 10 μM 2-AG: 4.8 μM (3.4 – 6.8)	479.6 % (436.3 – 527.4)	X. laevis oocytes	(Bakas et al. 2017)
GlyR	NAM		I/ICTRL(*100%) 40 ±7% 1 μM	hippocampal CA1 and CA3 pyramidal neurons and Purkinje neurons	(Lozovaya et al. 2005)
GlyR α1	NAM			CHO cells	(Lozovaya et al. 2011)
nAChR α7	NAM	IC50 168 nM		X. laevis oocytes	(Oz et al. 2004)

Noladineether (NE)
2-AGE

Receptor	Effects	Results	max. potentiation / max. inhibition	System	Source
GABAAR α1β2γ2	PAM	~65 % compared to 2-AG		X. laevis oocytes	(Sigel et al. 2011)

N-Arachidonoyl Serine
(NASer)

Receptor	Effects	Results	max. potentiation / max. inhibition	System	Source
GABAAR α1β2γ2	PAM	~200 % compared to 2-AG		X. laevis oocytes	(Baur et al. 2013)
GlyR α1	PAM			HEK293 cells	(Yévenes and Zeilhofer 2011)
GlyR α2	NAM			HEK293 cells	(Yévenes and Zeilhofer 2011)
GlyR α3	NAM			HEK293 cells	(Yévenes and Zeilhofer 2011)

N-arachidonyl-glycine
(NAGly)

Receptor	Effects	Results	max. potentiation / max. inhibition	System	Source
GABAAR α1β2γ2	PAM		~300 % compared to 2-AG	X. laevis oocytes	(Baur et al. 2013)
GABAAR α1β2γ2	PAM		~450 % at 3 μM NA-Gly	X. laevis oocytes	(Baur et al. 2013)
GABAAR α1β1γ2	PAM		~40 % at 3 μM NA-Gly	X. laevis oocytes	(Baur et al. 2013)

GlyR α1	NAM	IC50 2,4 µM ± 0.7 µM at EC80<br>IC50 24 µM ± 6,2 µM at EC10<br>“bell-shaped”		HEK293 cells	(Yang et al. 2008)
GlyR α2	NAM	IC50 3,03 µM ± 0,09 µM		HEK293 cells	(Yang et al. 2008)
GlyR α3	NAM	IC50 1.32 µM 0.10 µM		HEK293 cells	(Yang et al. 2008)
GlyR α1	PAM		101 ± 11% at 10 µM NA-Gly	HEK293 cells	(Yévenes and Zeilhofer 2011)
GlyR α2	NAM		-56 ± 5% at 10 µM NA-G	HEK293 cells	(Yévenes and Zeilhofer 2011)
GlyR α3	NAM		-32 ± 3% at 10 µM NA-G	HEK293 cells	(Yévenes and Zeilhofer 2011)

Tetrahydrocannabinol (THC)

Receptor	Effects	Results	max. potentiation / max. inhibition	System	Source
GABAAR α1β2γ2	PAM	~30 % compared to 2-AG at 3 µM THC		X. laevis oocytes	(Sigel et al. 2011)
GABAAR α1β2γ2	PAM	THC at 3 µM significantly enhanced GABA-activated currents		HEK293 cells	(Yao et al. 2020)
GABAAR α2β3γ2	-	no effects at 300 nM THC		X. laevis oocytes	(Hejazi et al. 2006)
GABAAR α2β3γ2	PAM	weak effects at 10 µM THC***		X. laevis oocytes	(Schmiedhofer 2017)
GABAAR α4β1δ	NAM	GABA EC50 0,02 µM (0,01 - 0,02)<br>GABA + 10 µM THC EC50 1,2 (0,20 - 6,66)***		X. laevis oocytes	(Schmiedhofer 2017)
GABAAR α4β3δ	PAM	GABA EC50 3,0 µM (2,42 - 3,72)<br>GABA + 10 µM THC EC50 0,4 µM (0,19 - 0,99)***		X. laevis oocytes	(Schmiedhofer 2017)
GABAAR α6β3	PAM		~500% at 1 µM THC***	X. laevis oocytes	(Schmiedhofer 2017)
GABAAR α4β3	PAM		~300% at 1 µM THC***	X. laevis oocytes	(Schmiedhofer 2017)
GABAAR α2β3	PAM	GABA EC50 6,0 µM<br>GABA + 10 µM THC EC50 1,39 µM***		X. laevis oocytes	(Schmiedhofer 2017)
GABAAR α2β3	PAM	GABA EC50 10,6 µM<br>GABA + 10 µM THC EC50 3,7 µM***		X. laevis oocytes	(Schmiedhofer 2017)
GlyR α1β	PAM		44 ± 13% at 30 nM THC<br>82 ± 4% at 100 nM THC 136 ± 11% at 300 nM THC	cultured spinal neurons	(Xiong et al. 2011)
GlyR α2	PAM		232 ± 35% at 1 µM THC	HEK293 cells	(Xiong et al. 2011)
GlyR α3	PAM		97 ± 7% at 100 nM THC<br>1127 ± 142% at 1 µM THC	HEK293 cells	(Xiong et al. 2011)
GlyR α1	PAM		1156 ± 472% 1 µM THC	HEK293 cells	(Xiong et al. 2011)

GlyR α1	PAM		~1000 % at 1 µM THC****	HEK293 cells	(Yao et al. 2020)
GlyR α3	PAM		~1000 % at 1 µM THC****	HEK293 cells	(Yao et al. 2020)
GlyR α1β	PAM		~400 % at 1 µM THC****	HEK293 cell	(Yao et al. 2020)
GlyR α1	PAM	162 % ± 12 % at 1 µM THC EC50 1.3 µM ± 0.6 µM	260% ± 30 % at max.	X. laevis oocytes	(Wells et al. 2015)
5HT3A	NAM	THC at 1 µM significantly reduced the 5-HT-activated current		HEK293 cells	(Yao et al. 2020)
5HT3A	NAM	IC50 38,4 nM		HEK293 cells	(Barann et al. 2002)
5HT3A	NAM	IC50 119 nM ±13 nM	97 % ± 5% at 1 µM THC	HEK293 cells	(Xiong et al. 2011)
5HT3A	NAM	IC50 285 nm ± 23 nM 1 ng of cRNA IC50 1.2 µM ± 0.3 µM 3 ng of cRNA.	68,5% at 3 µM THC with 3 ng cRNA	X. laevis oocytes	(Yang et al. 2010)
nACh α7	-	10 µM THC show no effect		X. laevis oocytes	(Mahgoub et al. 2013)
nAChR α4β2	-	THC has no impact, even coapplied wit AEA		SH-EP1 cells	(Spivak et al. 2007)
nAChR α7	-	THC has no impact		X. laevis oocytes	(Oz et al. 2004)

Cannabidiol (CBD)

Receptor	Effects	Results	max. potentiation / max. inhibition	System	Source
GlyR α3	PAM		491 ± 101% at 1 µM CBD	HEK293 cells	(Xiong et al. 2012)
GlyR α1β	PAM	EC50 Glycine + CBD: 12.3 ± 3.8 µmol/l EC50 Glycine: 132.4 ± 12.3 µmol/l		HEK293 cells	(Ahrens et al. 2009)
GlyR α1	PAM	EC50 Glycine + CBD: 18.1 ± 6.2 µmol/l EC50 Glycine: 144.3 ± 22.7 µmol/l		HEK293 cells	(Ahrens et al. 2009)
GlyR α1 S267I	-	S267I in TM2 diminishes CBD modulatory effects of GlyR		HEK293 cells	(Foadi et al. 2010)
GABAAR α1β2	PAM	EC50 GABA: 5.6 µM (3.6 – 6.9) EC50 GABA + 10 µM CBD: 3.7 µM (1.7 – 8.0)	217.3 % (174.7 – 259.9)	X. laevis oocytes	(Bakas et al. 2017)
GABAAR α1β2γ2L	PAM	EC50 GABA: 181.3 µM (127.0 – 258.7) EC50 GABA + 10 µM CBD: 6.5 µM (3.5 – 12.3)	153.9 % (132.6 – 175.1)	X. laevis oocytes	(Bakas et al. 2017)
GABAAR α2β2	PAM	EC50 GABA: 22.9 µM (15.4 – 34.1) EC50 GABA + 10 µM CBD: 2.0 µM (1.0 – 4.1)	262.6 % (219.2 – 305.9)	X. laevis oocytes	(Bakas et al. 2017)
GABAAR α2β2γ2L	PAM	EC50 GABA: 214.5 µM (157.7 – 255.3) EC50 GABA + 10 µM CBD: 3.7 µM (1.7 – 8.0)	331.5 % (277.2 – 391.1)	X. laevis oocytes	(Bakas et al. 2017)

GABAAR α2β2(V436T)γ2L	PAM	EC50 GABA: 219.2 μM (185.5 – 259.0)<br>EC50 GABA + 10μM CBD: 13.3 μM (4.6 – 35.8)	123.6 % (79.9 – 167.2)	X. laevis oocytes	(Bakas et al. 2017)
GABAAR α3β2γ2L	PAM	EC50 GABA: 155.1 μM (96.1 – 250.6)<br>EC50 GABA + 10 μM CBD: 10.0 μM (6.0 – 16.7)	129.4 % (109.9 – 148.8)	X. laevis oocytes	(Bakas et al. 2017)
GABAAR α4β2γ2L	PAM	EC50 GABA: 84.6 μM (62.7 – 114.1)<br>EC50 GABA + 10 μM CBD: 0.9 μM (0.4 – 1.8)	90.4 % (74.8 – 105.9)	X. laevis oocytes	(Bakas et al. 2017)
GABAAR α5β2γ2L	PAM	EC50 GABA: 24.2 μM (21.4 – 27.3)<br>EC50 GABA + 10 μM CBD: 1.4 μM (0.6 – 3.2)	73.6 % (57.0 – 90.3)	X. laevis oocytes	(Bakas et al. 2017)
GABAAR α6β2γ2L	PAM	EC50 GABA: 13.4 μM (7.5 – 24.3)<br>EC50 GABA + 10 μM CBD: 8.2 μM (2.4 – 28.1)	71.9 % (42.6 – 101.2)	X. laevis oocytes	(Bakas et al. 2017)
GABAAR α2β1γ2L	PAM	EC50 GABA: 142.2 μM (105.4 – 191.7)<br>EC50 GABA + 10 μM CBD: 17.4 μM (9.4 – 23.0)	151.4 % (123.5 – 189.9)	X. laevis oocytes	(Bakas et al. 2017)
GABAAR α2β3γ2L	PAM	EC50 GABA: 50.8 μM (32.7 – 78.9)<br>EC50 GABA + 10 μM CBD: 4.4 μM (3.1– 6.3)	268.4 % (249.6 – 287.6)	X. laevis oocytes	(Bakas et al. 2017)
GABAAR α4β2δ	PAM	EC50 GABA + 10 μM CBD: 23.1 μM (15.8 – 33.7)	752.4 % (649.2 – 881.5)	X. laevis oocytes	(Bakas et al. 2017)
GABAAR α4β3δ	PAM	EC50 GABA: 3,0 μM (2,42 - 3,72)<br>EC50 GABA + CBD: 0.41 μM (0,10 - 1,69)	1170 % ± 127.2 at 10 μM CBD at EC 3-5 GABA<br><br>259.7 % ± 17.42 at 10 μM CBD at EC 90-100 GABA	X. laevis oocytes	(Schmiedhofer 2017)
GABAAR α4β1δ	NAM	EC50 GABA + 10 μM CBD 0.034μM***<br>EC50 GABA 0.015μM***		X. laevis oocytes	(Schmiedhofer 2017)
GABAAR α6β3δ	PAM	EC50 GABA + 10 μM CBD EC50 1.2μM (0,16 - 8,65)<br>EC50 GABA 3.16μM (1,91 - 5,23)		X. laevis oocytes	(Schmiedhofer 2017)
5HT3A	NAM	IC50 329 nM ±19 nM	82 % ± 13% at 1 μM CBD	HEK293 cells	(Xiong et al. 2011)
5HT3A	NAM	IC50 0.6 μM ± 0.1 μM	81% ± 5% at 1 μM CBD injected with 1 ng of 5-HT3A receptor cRNA	X. laevis oocytes	(Yang et al. 2010)
nAChR α7	NAM	IC50 11.3 μM ± 1.8 μM		Whole-cell patch clamp recordings in rat hippocampal slices	(Mahgoub et al. 2013)

DH-Cannabidiol (DH-CBD)

Receptor	Effects	Results	max. potentiation / max. inhibition	System	Source
GlyR α1	PAM	DH-CBD significantly reduced glycine EC50 values in seven of nine hyperekplexia mutant receptors		HEK293 cells	(Xiong et al. 2014)

GlyR α1 R271Q	PAM	EC50 Glycine: 21 μM ± 3.2 μM EC50 Glycine + DH-CBD: 1.2 μM ± 0.1 μM		HEK293 cells	(Xiong et al. 2014)
GlyR α3	PAM	EC50 Glycine: 377 μM ± 45 μM EC50 Glycine + DH-CBD: 195 μM ± 51 μM at 1 μM DH-CBD EC50 Glycine + DH-CBD: 58 μM ± 13 μM at 10 μM DH-CBD	989 ± 171% at 1 μM DH-CBD	HEK293 cells	(Xiong et al. 2012)
GlyR α1	PAM	DH-CBD at 1 mM remarkably enhanced IGly		HEK293 cells	(Lu et al. 2018)
GABAAR α1β2γ2	-	no effect on GABAR alone, DH-CBD-induced disruption of the interaction between GlyR α1 R271Q and GABAAR		HEK293 cells	(Zou et al. 2020)

Cannabigerol (CBG)

Receptor	Effects	Results	max. potentiation / max. inhibition	System	Source
GABAAR α1β3γ2	PAM	EC50 GABA: 21.3 μM (18,67 - 24,4) *** EC50 GABA + 10μM CBG: 12.49 μM (3,83 - 40,79) ***	~200 % ***	X. laevis oocytes	(Schmiedhofer 2017)
GABAAR α6β3δ	PAM		~300 % ***	X. laevis oocytes	(Schmiedhofer 2017)
GABAAR α6β3γ2	PAM	EC50 GABA: 3.638 μM (2,44 - 5,42) *** EC50 GABA + 10 μM CBG: 3.084μM (0,97 - 9,77 μM) ***	~150 % ***	X. laevis oocytes	(Schmiedhofer 2017)

Ajulemic Acid

Receptor	Effects	Results	max. potentiation / max. inhibition	System	Source
GlyR α1 S267I	-			HEK293 cells	(Foadi et al. 2010)

beta-Caryophyllen (BCP)

Receptor	Effects	Results	max. potentiation / max. inhibition	System	Source
GABAAR α4β3δ	NAM		80 % ± 33 at 600 μM**	HEK293 cells	(Janzen et al. 2021)
GABAAR α6β3δ	NAM		76 % ± 26 at 600 μM**	HEK293 cells	(Janzen et al. 2021)
GABAAR α1β2γ2	NAM		90 % ± 37 at 600 μM**	HEK293 cells	(Janzen et al. 2021)
GABAAR α1β2	NAM		98 % ± 13 at 600 μM**	HEK293 cells	(Janzen et al. 2021)

HU-210

Receptor	Effects	Results	max. potentiation / max. inhibition	System	Source
GlyR α1	PAM	EC50 co-activation: 5.1 μM ± 2.6<br>EC50 direct activation: 188.7 μM ± 46.2		HEK293 cells	(Demir et al. 2009)
GlyR α1 S267I	-			HEK293 cells	(Foadi et al. 2010)
GlyR α1	PAM	EC50: 0.27 μM ± 0.05		HEK293 cells	(Yang et al. 2008)
GlyR α1β	PAM		78 % ± 5 at 30 μM	HEK293 cells	(Yang et al. 2008)
GlyR α2	NAM	IC50: 0.090 μM ± 0.021		HEK293 cells	(Yang et al. 2008)
GlyR α3	NAM	IC50: 0.050 μM ± 0.006		HEK293 cells	(Yang et al. 2008)

Rimonabant (SR141716A)

Receptor	Effects	Results	max. potentiation / max. inhibition	System	Source
GABAAR α1β2γ2	PAM	EC50: 7.3 μM ± 0.5 μM	3381 % ± 165	X. laevis oocytes	(Baur et al. 2012)

AM251

Receptor	Effects	Results	max. potentiation / max. inhibition	System	Source
GABAAR α1β2γ2	PAM	EC50 0.40 μM ± 0.13<br><br>EC50 GABA: 15.4 μM ± 0.8 μM<br>EC50 GABA + 1μM AM251: 5.5 μM ± 0.4 μM	881 % ± 167 at 3 μM AM251 + 0,5 μM GABA	X. laevis oocytes	(Baur et al. 2012)
GABAAR α1β2	PAM		~550 % at 3 μM AM251 + 0,5 μM GABA*	X. laevis oocytes	(Baur et al. 2012)
GABAAR α1β3γ2	PAM		~1250 % at 3 μM AM251 + 0,5 μM GABA*	X. laevis oocytes	(Baur et al. 2012)
GABAAR α2β2γ2	PAM		~850 % at 3 μM AM251 + 0,5 μM GABA*	X. laevis oocytes	(Baur et al. 2012)
GABAAR α3β2γ2	PAM		~400 % at 3 μM AM251 + 0,5 μM GABA*	X. laevis oocytes	(Baur et al. 2012)
GABAAR α5β2γ2	PAM		~500 % at 3 μM AM251 + 0,5 μM GABA*	X. laevis oocytes	(Baur et al. 2012)
GABAAR α6β2γ2	PAM		~200 % at 3 μM AM251 + 0,5 μM GABA*	X. laevis oocytes	(Baur et al. 2012)
GABAAR α1β1γ2	PAM		~20 % at 3 μM AM251 + 0,5 μM GABA*	X. laevis oocytes	(Baur et al. 2012)

GABAAR α4β2δ	PAM		~350 % at 3 µM AM251 + 0,5 µM GABA*	X. laevis oocytes	(Baur et al. 2012)

WIN55,212-2

Receptor	Effects	Results	max. potentiation / max. inhibition	System	
GlyR α1	-			HEK293 cells	(Yang et al. 2008)
GlyR α1β	-			HEK293 cells	(Yang et al. 2008)
GlyR α2	NAM	IC50: 0.22 µM ± 0.05		HEK293 cells	(Yang et al. 2008)
GlyR α3	NAM	IC50: 0.050 µM ± 0.006		HEK293 cells	(Yang et al. 2008)
5HT3A	NAM	IC50: 103.5 nM		HEK293 cells	(Barann et al. 2002)
nAChR α7	-	no effects		X. laevis oocytes	(Oz et al. 2004)

*read from graph
**high concentration of substance
*** low sample size (n < 4)
**** Δ9-THC effects are cholesterol dependent

Supplementary Table 2. Cys-loop receptors genetic variants in epilepsies. Cys-loop receptors genetic variants in epilepsies. This table provides an overview of variants in Cys-loop receptor subunit encoding genes identified in patients.

**Epilepsy syndrom**	**genetic basis**
DS (Dravet syndrome)	GABRA1 (S76R, R112Q, L146M, R214C, R214H, L215P, G251S, V287I, K306T<br>GABRB2 (A159S, Y181F, F331l, F331S)<br>GABRB3 (T157M, R232Q, T281I)<br>GABRG2 (Q40X, T90R, P302L, Q390X)<br>(Fu et al., 2022)
LGS (Lennox gastaut syndrome)	GABRB3 (D120N) (Qu et al., 2020)<br>GABRB3(D120N, E180G, Y302C) (Janve et al., 2016)<br>GABRA1 (T292I)<br>GABRB2 (I246T, P252L, I288S)<br>GABRB3 (D120N, E180G, Y302C, A305T, N328D)<br>GABRG2 (P83S) (Fu et al., 2022)
WS (West syndrome) and IS (infantile spasm)	GABRA1 (R112Q, P260S, P260L, M263T, M263I, T292I, L296S, W315L)<br>GABRB2 (T184I, R240T, F245S, P252L, I299S)<br>GABRB3 (L52V, I69T, E77K, M80L, N110D, L256Q, L278F, Y302C)<br>(Fu et al., 2022)<br>GABRB3(N110D, E180G)<br>GABRB1(F246S) (Janve et al., 2016)
EIEE (early infantile epileptic encephalopathy)	GABRA1 (P260L, T289P, T289A)<br>GABRB2 (K303N)<br>GABRB3 (T287I) (Fu et al., 2022)
EMA (early myoclonic encephalopathy)	GABRB2 (I246T, T284K, T287P) (Fu et al., 2022)
EOEE (early onset epileptic encephalopathy)	GABRA2 (T292K) (Butler et al., 2018)<br>GABRA1 (R112Q, N115D, V287L, A332V)<br>GABRB2 (P252L, K298G, K303R)<br>GABRB3 (N110D, K127R, L170R, T185I, S254F, L256Q, T288N, L293H, A305V) (Fu et al., 2022)
EIMFS (epilepsy of infancy with migrating focal seizures)	GABRA1 (P280T)<br>GABRB3 (L124F, Y245H, S254F, T281A, L284M)
CAE (Childhood absence epilepsy)<br>JAE (Juvenile absence epilepsy)	GABRB3 (G32R) (Gurba et al., 2012)<br>GABRA1 (R214C, L267I, S326fs328X)<br>GABRB2 (V316I)<br>GABRB3 (P11S, S15F, G32R, V37G, E357K)<br>GABRG2 (R82Q, T90M, R177fs) (Fu et al., 2022)
SHE (sleep-related hypermotor epilepsy)<br>ADSHE formerly autosomal dominant nocturnal frontal lobe epilepsy (ADNFLE)	GABRB2 V337G<br>CHRNA4 R336H<br>CHRNB2 V287L<br>CHRNA2 I279A<br>CHRNA4 S284L

	CHRNA4 S280P<br>CHRNA4 insL (Becchetti et al., 2020)
MAE (epilepsy with myoclonic-atonic seizures)<br>JME (juvenile myoclonic epilepsy)<br>MSE (myoclonic status epilepsy)	GABRA1 (F104C, R214C, K306T, A322D)<br>GABRB2 (V262F)<br>GABRB3 (S76C, R111X, D120N, R142L, Y184H)<br>GABRG2 (R323Q) (Fu et al., 2022)
IGE (idiopathic generalized epilepsies)	GABRA1 (A322D)<br>GABRA4 (T320A, L26M)<br>GABRA5 (I48L)<br>GABRA6 (R46W, P385S)<br>GABRP (V10M)<br>GABRE (G66S, Y38S)<br>GABRG2 (R43Q, K289M, Q351X)<br>GABRD (E177A, R220H) (Dibbens et al., 2009)
Unspecified EE	GABRA1 (G251D)<br>GABRB2 (M79T, D125N, Y244H, P252A, L277S, T287P, I288S)<br>GABRB3 (Y182F, R232X, R232Q, Q249K, P253L, P301L, Y302C)<br>GABRG2 (A106T, I107T, P282T, P282S, R323W, F343L) (Fu et al., 2022)
DEE (developmental and epileptic encephalopathy)<br>EDD (epileptogenic developmental disorders)<br>GDD (global development delay)<br>NDD (neurodevelopmental disorder)<br>NDDE (neurodevelopmental disorder with epilepsy)	GABRA1 (M79T, A112E, D125N, Y181F, Y183H, T184I, F224C, R240T, Y244H, F245S, P252A, P252T, V262F, L277S, V282A, T284K, R293P, K298G, Y301C, K303N, A304V)<br>GABRB1 (V78L, M80L, Q249H, P253S, T288I, L321P) (Fu et al., 2022)
Generalized epilepsy with febrile seizures	GABRD (G177A) variant (Dibbens et al., 2004)<br>GABRA1 (V74I9)<br>GABRB2 (D108Y, V133M, M161L, N350_del)<br>GABRB3 (P54L, T157M, R429Q)<br>GABRG2 (Q40X, N79S, P83S, T90M, R136X, R323Q, K328M, Q390X, W429X) (Fu et al., 2022)
FS (febrile seizure)	GABRB2 (R354C)<br>GABRB3 (T157M)<br>GABRG2 (R82Q, R136X, R177G, R177fs, K328M) (Fu et al., 2022)
RE (rolandic epilepsy)	GABRG2 (G257R, R323Q, I389V) (Fu et al., 2022)
Complex epilepsy	GABRD (E177A, R220H) (Feng et al., 2006)

Development of epilepsy	GABRA1 (A294D) mutation (Fisher, 2004)

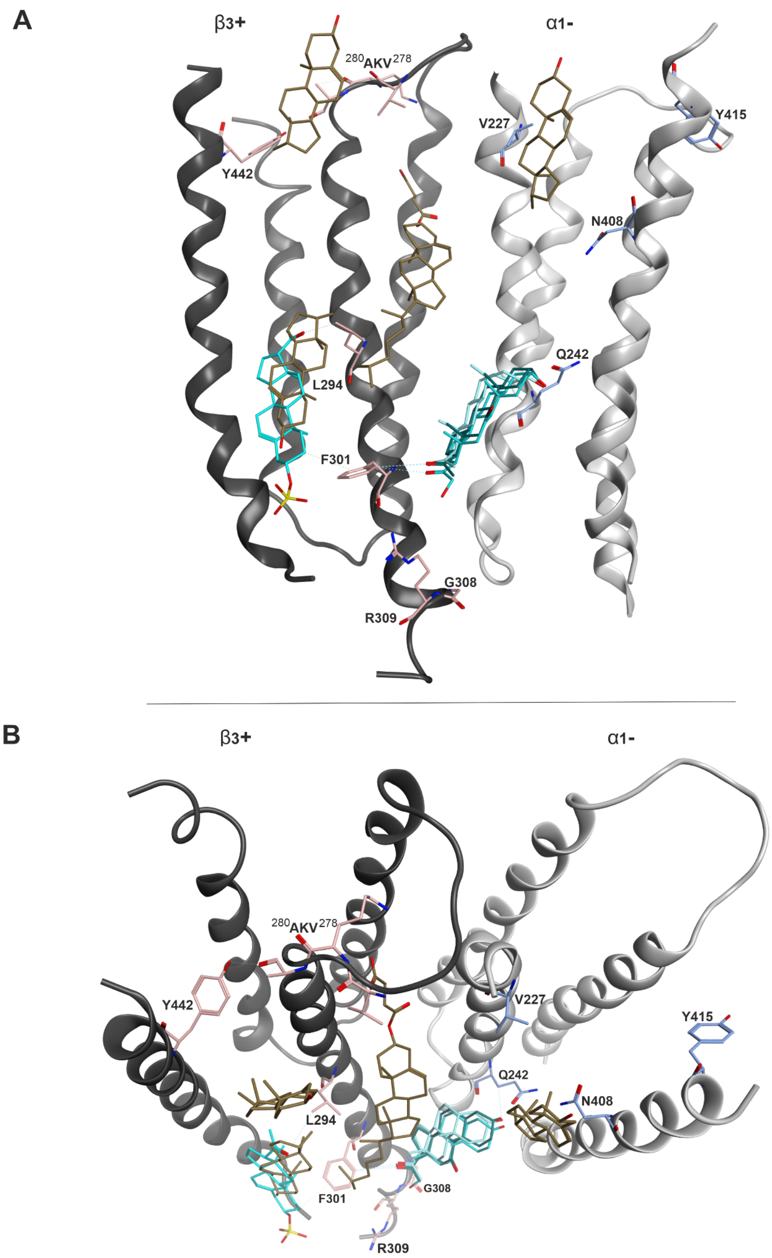

Supplementary Figure 1. Steroid binding sites on a β3+ (dark grey) α1- (light grey) $GABA_A$ receptor dimer (PDB ID 6HUO - Masiulis et al., 2019). Amino acids which have been proposed based on photoaffinity labeling by Chen et al., 2019 are rendered in stick representation. Color coding: Steroid binding site at the lower interface site:
THDOC (5OSB in cyan - Laverty et al., 2017), alphaxalone (6CDU in dark cyan - Chen et al., 2018), pregnanolone (5OJM in light cyan - Miller et al., 2017); lipid associated lower TMD site: pregnenolone sulfate (5OSC in cyan - Laverty et al., 2017) and cholesterol (6D6T in brown - Zhu et al., 2018); lipid associated upper TMD site at the β3+ subunit: cholesterol (6D6T in brown); lipid associated upper TMD site at the α1- subunit: cholesterol (6D6T in brown); lipid associated TMD interface site: cholesterol hemisuccinate (5OSC in brown - Laverty et al., 2017)

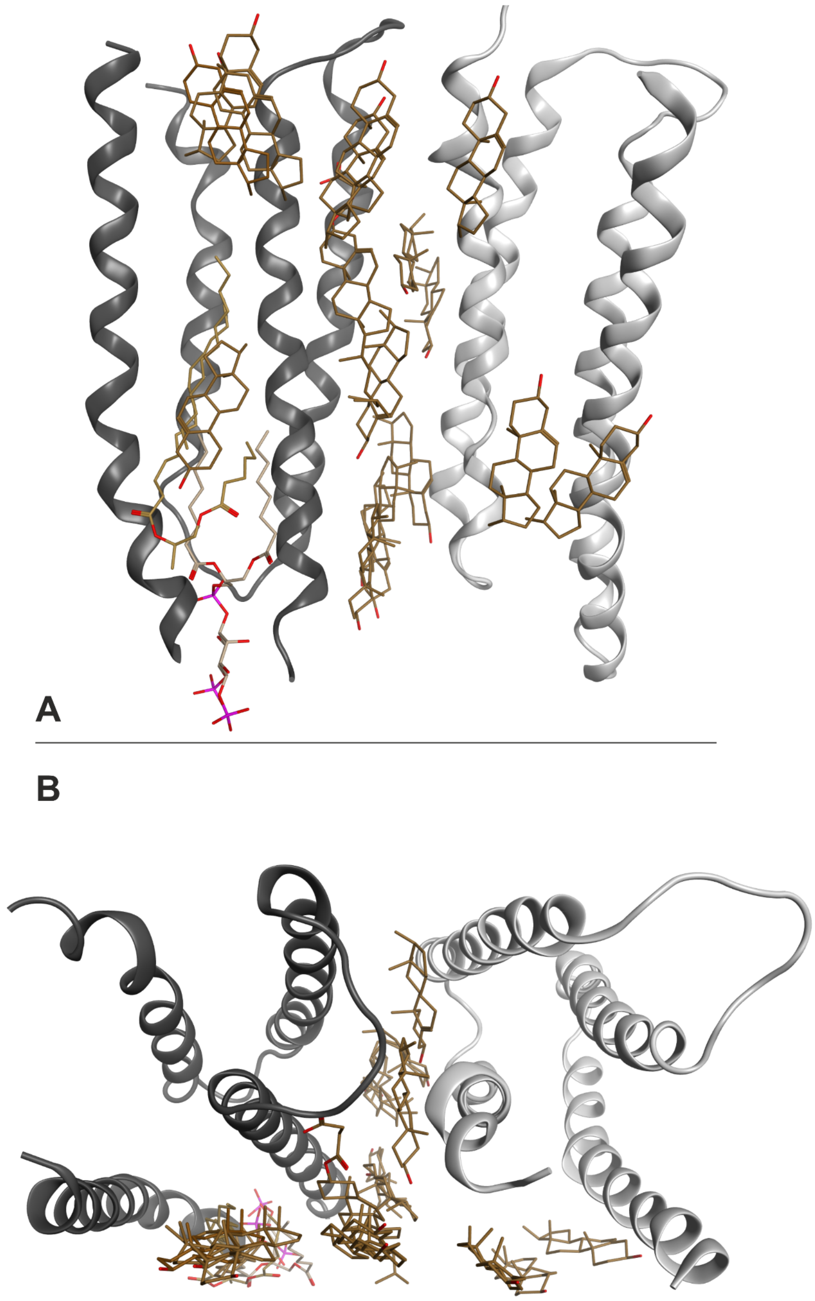

Supplementary Figure 2. Cholesterol, cholesterol derivatives and other endogenous lipid molecules: PDB files with models of cholesterol (and derivatives), and PIP2 (6I53 - Laverty et al., 2019, 6D6T, 6D6U and 5OSC) have been superposed to render the localizations of the lipid molecules in direct comparison. A: Dimer seen from the outside. B: dimer seen from the perspective of the ECD. Dark grey ribbon: principal subunit, light grey ribbon: complementary subunit. Ribbon rendering of 6D6U. All cholesterols are from 6D6U and 6D6T. The cholesterol hemisuccinate at the interface site is from 5OSC.

Pilot experiment: plant matrix vs. extract fractions

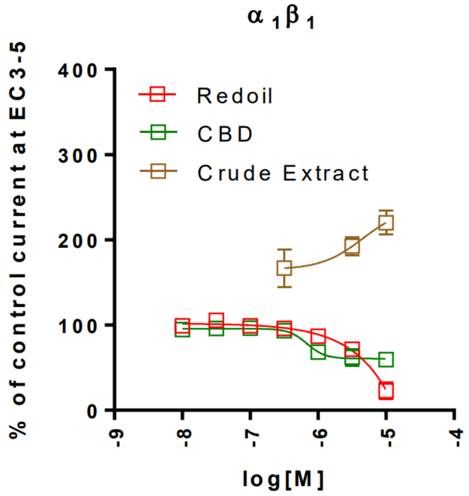

Supplementary Figure 3. Pilot Experiment. This Figure shows distinct effects of cannabis extract fractions on a1b1 GABAAR. **Crude extract** produced with CO2 extraction contains most substances from cannabis, including the cannabinoid part (25% CBD, 4% Δ9-THC). **Redoil** a fraction of the crude extract consists of a fraction containing mostly cannabinoids(~90% CBD, ~10% Δ9-THC, and other plant compounds), **CBD** was used in purified form (99,87 %, BSPG, UK) The Figure was adopted from (Schmiedhofer, 2017).

References used only in the Supplementary Materials

Ahrens, J., Demir, R., Leuwer, M., de la Roche, J., Krampfl, K., Foadi, N., Karst, M., and Haeseler, G. (2009). The nonpsychotropic cannabinoid cannabidiol modulates and directly activates alpha-1 and alpha-1-Beta glycine receptor function. *Pharmacology* 83, 217–222. doi:10.1159/000201556.

Bakas, T., van Nieuwenhuijzen, P. S., Devenish, S. O., McGregor, I. S., Arnold, J. C., and Chebib, M. (2017). The direct actions of cannabidiol and 2-arachidonoyl glycerol at GABAA receptors. *Pharmacol. Res.* 119, 358–370. doi:10.1016/j.phrs.2017.02.022.

Barann, M., Molderings, G., Brüss, M., Bönisch, H., Urban, B. W., and Göthert, M. (2002). Direct inhibition by cannabinoids of human 5-HT3A receptors: probable involvement of an allosteric modulatory site. *Br. J. Pharmacol.* 137, 589–596. doi:10.1038/sj.bjp.0704829.

Baur, R., Gertsch, J., and Sigel, E. (2013a). Do N-arachidonyl-glycine (NA-glycine) and 2-arachidonoyl glycerol (2-AG) share mode of action and the binding site on the β2 subunit of

GABAA receptors? *PeerJ* 1, e149. doi:10.7717/peerj.149.

Baur, R., Gertsch, J., and Sigel, E. (2012). The cannabinoid CB1 receptor antagonists rimonabant (SR141716) and AM251 directly potentiate GABA(A) receptors. *Br. J. Pharmacol.* 165, 2479–2484. doi:10.1111/j.1476-5381.2011.01405.x.

Baur, R., Kielar, M., Richter, L., Ernst, M., Ecker, G. F., and Sigel, E. (2013b). Molecular analysis of the site for 2-arachidonylglycerol (2-AG) on the β_2 subunit of GABA(A) receptors. *J. Neurochem.* 126, 29–36. doi:10.1111/jnc.12270.

Becchetti, A., Grandi, L. C., Colombo, G., Meneghini, S., and Amadeo, A. (2020). Nicotinic Receptors in Sleep-Related Hypermotor Epilepsy: Pathophysiology and Pharmacology. *Brain Sci.* 10. doi:10.3390/brainsci10120907.

Butler, K. M., Moody, O. A., Schuler, E., Coryell, J., Alexander, J. J., Jenkins, A., and Escayg, A. (2018). De novo variants in GABRA2 and GABRA5 alter receptor function and contribute to early-onset epilepsy. *Brain* 141, 2392–2405. doi:10.1093/brain/awy171.

Butt, C., Alptekin, A., Shippenberg, T., and Oz, M. (2008). Endogenous cannabinoid anandamide inhibits nicotinic acetylcholine receptor function in mouse thalamic synaptosomes. *J. Neurochem.* 105, 1235–1243. doi:10.1111/j.1471-4159.2008.05225.x.

Chen, Q., Wells, M.M., Arjunan, P. et al. Structural basis of neurosteroid anesthetic action on GABAA receptors. Nat Commun 9, 3972 (2018). https://doi.org/10.1038/s41467-018-06361-4

Chen ZW, Bracamontes JR, Budelier MM, Germann AL, Shin DJ, et al. (2019) Multiple functional neurosteroid binding sites on GABAA receptors. PLOS Biology 17(3): e3000157. https://doi.org/10.1371/journal.pbio.3000157

Demir, R., Leuwer, M., de la Roche, J., Krampfl, K., Foadi, N., Karst, M., Dengler, R., Haeseler, G., and Ahrens, J. (2009). Modulation of glycine receptor function by the synthetic cannabinoid HU210. *Pharmacology* 83, 270–274. doi:10.1159/000209291.

Dibbens, L. M., Feng, H.-J., Richards, M. C., Harkin, L. A., Hodgson, B. L., Scott, D., Jenkins, M., Petrou, S., Sutherland, G. R., Scheffer, I. E., et al. (2004). GABRD encoding a protein for extra- or peri-synaptic GABAA receptors is a susceptibility locus for generalized epilepsies. *Hum. Mol. Genet.* 13, 1315–1319. doi:10.1093/hmg/ddh146.

Dibbens, L. M., Harkin, L. A., Richards, M., Hodgson, B. L., Clarke, A. L., Petrou, S., Scheffer, I. E., Berkovic, S. F., and Mulley, J. C. (2009). The role of neuronal GABA(A) receptor subunit mutations in idiopathic generalized epilepsies. *Neurosci. Lett.* 453, 162–165. doi:10.1016/j.neulet.2009.02.038.

Feng, H.-J., Kang, J.-Q., Song, L., Dibbens, L., Mulley, J., and Macdonald, R. L. (2006). Delta subunit susceptibility variants E177A and R220H associated with complex epilepsy alter channel gating and surface expression of alpha4beta2delta GABAA receptors. *J. Neurosci.* 26, 1499–1506. doi:10.1523/JNEUROSCI.2913-05.2006.

Fisher, J. L. (2004). A mutation in the GABAA receptor alpha 1 subunit linked to human epilepsy affects channel gating properties. *Neuropharmacology* 46, 629–637.

doi:10.1016/j.neuropharm.2003.11.015.

Foadi, N., Leuwer, M., Demir, R., Dengler, R., Buchholz, V., de la Roche, J., Karst, M., Haeseler, G., and Ahrens, J. (2010). Lack of positive allosteric modulation of mutated alpha(1)S267I glycine receptors by cannabinoids. *Naunyn Schmiedebergs Arch Pharmacol* 381, 477–482. doi:10.1007/s00210-010-0506-9.

Fu, X., Wang, Y.-J., Kang, J.-Q., and Mu, T.-W. (2022). "GABAA receptor variants in epilepsy," in *Epilepsy*, ed. S. J. Czuczwar (Brisbane (AU): Exon Publications). doi:10.36255/exon-publications-epilepsy-gaba-receptor.

Gurba, K. N., Hernandez, C. C., Hu, N., and Macdonald, R. L. (2012). GABRB3 mutation, G32R, associated with childhood absence epilepsy alters α1β3γ2L γ-aminobutyric acid type A (GABAA) receptor expression and channel gating. *J. Biol. Chem.* 287, 12083–12097. doi:10.1074/jbc.M111.332528.

Hejazi, N., Zhou, C., Oz, M., Sun, H., Ye, J. H., and Zhang, L. (2006). Delta9-tetrahydrocannabinol and endogenous cannabinoid anandamide directly potentiate the function of glycine receptors. *Mol. Pharmacol.* 69, 991–997. doi:10.1124/mol.105.019174.

Janve, V. S., Hernandez, C. C., Verdier, K. M., Hu, N., and Macdonald, R. L. (2016). Epileptic encephalopathy de novo GABRB mutations impair γ-aminobutyric acid type A receptor function. *Ann. Neurol.* 79, 806–825. doi:10.1002/ana.24631.

Janzen, D., Slavik, B., Zehe, M., Sotriffer, C., Loos, H. M., Buettner, A., and Villmann, C. (2021). Sesquiterpenes and sesquiterpenoids harbor modulatory allosteric potential and affect inhibitory GABAA receptor function in vitro. *J. Neurochem.* 159, 101–115. doi:10.1111/jnc.15469.

Kimura, T., Ohta, T., Watanabe, K., Yoshimura, H., and Yamamoto, I. (1998). Anandamide, an endogenous cannabinoid receptor ligand, also interacts with 5-hydroxytryptamine (5-HT) receptor. *Biol. Pharm. Bull.* 21, 224–226. doi:10.1248/bpb.21.224.

Laverty, D., Thomas, P., Field, M. et al. Crystal structures of a GABAA-receptor chimera reveal new endogenous neurosteroid-binding sites. Nat Struct Mol Biol 24, 977–985 (2017). https://doi.org/10.1038/nsmb.3477

Laverty, D., Desai, R., Uchański, T. et al. Cryo-EM structure of the human α1β3γ2 GABAA receptor in a lipid bilayer. Nature 565, 516–520 (2019). https://doi.org/10.1038/s41586-018-0833-4

Lozovaya, N., Mukhtarov, M., Tsintsadze, T., Ledent, C., Burnashev, N., and Bregestovski, P. (2011). Frequency-Dependent Cannabinoid Receptor-Independent Modulation of Glycine Receptors by Endocannabinoid 2-AG. *Front. Mol. Neurosci.* 4, 13. doi:10.3389/fnmol.2011.00013.

Lozovaya, N., Yatsenko, N., Beketov, A., Tsintsadze, T., and Burnashev, N. (2005). Glycine receptors in CNS neurons as a target for nonretrograde action of cannabinoids. *J. Neurosci.* 25, 7499–7506. doi:10.1523/JNEUROSCI.0977-05.2005.

Lu, J., Fan, S., Zou, G., Hou, Y., Pan, T., Guo, W., Yao, L., Du, F., Homanics, G. E., Liu, D., et al. (2018). Involvement of glycine receptor α1 subunits in cannabinoid-induced analgesia.

Neuropharmacology 133, 224–232. doi:10.1016/j.neuropharm.2018.01.041.

Mahgoub, M., Keun-Hang, S. Y., Sydorenko, V., Ashoor, A., Kabbani, N., Al Kury, L., Sadek, B., Howarth, C. F., Isaev, D., Galadari, S., et al. (2013). Effects of cannabidiol on the function of α7-nicotinic acetylcholine receptors. *Eur. J. Pharmacol.* 720, 310–319. doi:10.1016/j.ejphar.2013.10.011.

Masiulis, S., Desai, R., Uchański, T. et al. GABAA receptor signalling mechanisms revealed by structural pharmacology. Nature 565, 454–459 (2019). https://doi.org/10.1038/s41586-018-0832-5

Miller, P., Scott, S., Masiulis, S. et al. Structural basis for GABAA receptor potentiation by neurosteroids. Nat Struct Mol Biol 24, 986–992 (2017). https://doi.org/10.1038/nsmb.3484

Oz, M., Ravindran, A., Diaz-Ruiz, O., Zhang, L., and Morales, M. (2003). The endogenous cannabinoid anandamide inhibits alpha7 nicotinic acetylcholine receptor-mediated responses in Xenopus oocytes. *J. Pharmacol. Exp. Ther.* 306, 1003–1010. doi:10.1124/jpet.103.049981.

Oz, M., Zhang, L., Ravindran, A., Morales, M., and Lupica, C. R. (2004). Differential effects of endogenous and synthetic cannabinoids on alpha7-nicotinic acetylcholine receptor-mediated responses in Xenopus Oocytes. *J. Pharmacol. Exp. Ther.* 310, 1152–1160. doi:10.1124/jpet.104.067751.

Qu, S., Catron, M., Zhou, C., Janve, V., Shen, W., Howe, R. K., and Macdonald, R. L. (2020). GABAA receptor β3 subunit mutation D120N causes Lennox-Gastaut syndrome in knock-in mice. *Brain Commun.* 2, fcaa028. doi:10.1093/braincomms/fcaa028.

Schmiedhofer, P. (2017). Pharmacology of GABAA receptor subtypes: Phytocannabinoids act on GABAA receptors.

Sigel, E., Baur, R., Rácz, I., Marazzi, J., Smart, T. G., Zimmer, A., and Gertsch, J. (2011). The major central endocannabinoid directly acts at GABA(A) receptors. *Proc Natl Acad Sci USA* 108, 18150–18155. doi:10.1073/pnas.1113444108.

Spivak, C. E., Lupica, C. R., and Oz, M. (2007). The endocannabinoid anandamide inhibits the function of alpha4beta2 nicotinic acetylcholine receptors. *Mol. Pharmacol.* 72, 1024–1032. doi:10.1124/mol.107.036939.

Wells, M. M., Tillman, T. S., Mowrey, D. D., Sun, T., Xu, Y., and Tang, P. (2015). Ensemble-based virtual screening for cannabinoid-like potentiators of the human glycine receptor α1 for the treatment of pain. *J. Med. Chem.* 58, 2958–2966. doi:10.1021/jm501873p.

Xiong, W., Cheng, K., Cui, T., Godlewski, G., Rice, K. C., Xu, Y., and Zhang, L. (2011a). Cannabinoid potentiation of glycine receptors contributes to cannabis-induced analgesia. *Nat. Chem. Biol.* 7, 296–303. doi:10.1038/nchembio.552.

Xiong, W., Chen, S.-R., He, L., Cheng, K., Zhao, Y.-L., Chen, H., Li, D.-P., Homanics, G. E., Peever, J., Rice, K. C., et al. (2014). Presynaptic glycine receptors as a potential therapeutic target for hyperekplexia disease. *Nat. Neurosci.* 17, 232–239. doi:10.1038/nn.3615.

Xiong, W., Cui, T., Cheng, K., Yang, F., Chen, S.-R., Willenbring, D., Guan, Y., Pan, H.-L., Ren, K., Xu, Y., et al. (2012a). Cannabinoids suppress inflammatory and neuropathic pain by targeting α3 glycine receptors. *J. Exp. Med.* 209, 1121–1134. doi:10.1084/jem.20120242.

Xiong, W., Hosoi, M., Koo, B.-N., and Zhang, L. (2008). Anandamide inhibition of 5-HT3A receptors varies with receptor density and desensitization. *Mol. Pharmacol.* 73, 314–322. doi:10.1124/mol.107.039149.

Xiong, W., Koo, B. N., Morton, R., and Zhang, L. (2011b). Psychotropic and nonpsychotropic cannabis derivatives inhibit human 5-HT(3A) receptors through a receptor desensitization-dependent mechanism. *Neuroscience* 184, 28–37. doi:10.1016/j.neuroscience.2011.03.066.

Xiong, W., Wu, X., Li, F., Cheng, K., Rice, K. C., Lovinger, D. M., and Zhang, L. (2012b). A common molecular basis for exogenous and endogenous cannabinoid potentiation of glycine receptors. *J. Neurosci.* 32, 5200–5208. doi:10.1523/JNEUROSCI.6347-11.2012.

Yang, K. H. S., Isaev, D., Morales, M., Petroianu, G., Galadari, S., and Oz, M. (2010). The effect of Δ9-tetrahydrocannabinol on 5-HT3 receptors depends on the current density. *Neuroscience* 171, 40–49. doi:10.1016/j.neuroscience.2010.08.044.

Yang, Z., Aubrey, K. R., Alroy, I., Harvey, R. J., Vandenberg, R. J., and Lynch, J. W. (2008). Subunit-specific modulation of glycine receptors by cannabinoids and N-arachidonyl-glycine. *Biochem. Pharmacol.* 76, 1014–1023. doi:10.1016/j.bcp.2008.07.037.

Yao, L., Wells, M., Wu, X., Xu, Y., Zhang, L., and Xiong, W. (2020). Membrane cholesterol dependence of cannabinoid modulation of glycine receptor. *FASEB J.* 34, 10920–10930. doi:10.1096/fj.201903093R.

Yévenes, G. E., and Zeilhofer, H. U. (2011). Molecular sites for the positive allosteric modulation of glycine receptors by endocannabinoids. *PLoS ONE* 6, e23886. doi:10.1371/journal.pone.0023886.

Zhu, S., Noviello, C.M., Teng, J. et al. Structure of a human synaptic GABAA receptor. Nature 559, 67–72 (2018). https://doi.org/10.1038/s41586-018-0255-3

Zou, G., Xia, J., Han, Q., Liu, D., and Xiong, W. (2020). The synthetic cannabinoid dehydroxylcannabidiol restores the function of a major GABAA receptor isoform in a cell model of hyperekplexia. *J. Biol. Chem.* 295, 138–145. doi:10.1074/jbc.RA119.011221.
